# Phosphorylation‐Induced Degradation of Pepper Dehydration‐Related Homeobox Gene 12 (CaDRHB12), a Negative Regulator of Drought Response in Pepper, by CaSnRK2.6 Enhances Drought Tolerance

**DOI:** 10.1111/pbi.70298

**Published:** 2025-08-06

**Authors:** Woonhee Baek, Yeongil Bae, Chae Woo Lim, Sung Chul Lee

**Affiliations:** ^1^ Department of Life Science (BK21 Program) Chung‐Ang University Seoul Korea

**Keywords:** drought stress, homeodomain transcription factor, kinase, pepper, phosphorylation, protein degradation

## Abstract

Homeodomain‐leucine zipper (HD‐ZIP) transcription factors, particularly subfamily I genes, are associated with drought stress responses. However, the functional roles of HD‐ZIP subfamily II genes, particularly in pepper ( *Capsicum annuum* ), remain inadequately studied, and their involvement in abscisic acid (ABA) signalling is not fully elucidated. In this study, we isolated the HD‐ZIP subgroup II gene CaDRHB12 ( *C. annuum* drought‐responsive homeobox gene 12), which shares sequence homology with CaHAT1, a previously identified positive regulator of drought tolerance. Unlike that of *CaHAT1*, *CaDRHB12* expression was significantly downregulated upon ABA and drought stress treatment. Functional analyses of CaDRHB12 in pepper and Arabidopsis plants revealed that CaDRHB12 negatively regulates ABA signalling and drought stress responses. Furthermore, CaDRHB12 interacted with CaSnRK2.6, an ABA signalling‐related kinase that associates with and phosphorylates CaHAT1. In vitro and in vivo kinase assays demonstrated that CaSnRK2.6 also directly phosphorylates CaDRHB12, promoting CaDRHB12 protein degradation. This degradation was not observed in CaDRHB12 phospho‐dead and CaSnRK2.6 kinase‐dead mutants. Additionally, the transcriptional activity of CaDRHB12 was influenced by its phosphorylation status and CaSnRK2.6 function, highlighting the critical role of CaSnRK2.6‐mediated phosphorylation in regulating CaDRHB12 protein. Collectively, our findings suggest that CaDRHB12 negatively regulates pepper drought tolerance, and its protein stability is modulated by CaSnRK2.6‐mediated phosphorylation under drought stress conditions. Understanding the role of CaDRHB12 in drought stress response aids in developing strategies to improve plant resilience to abiotic stresses, potentially leading to more robust crops.

## 1 Introduction

During their life cycle, plants frequently face numerous abiotic stresses, including drought and osmotic stress, which result in water deficits and significantly limit their growth, development, and productivity. To cope with such adverse environmental conditions, plants have evolved complex mechanisms to enhance their resilience and facilitate adaptation (Zhang et al. 2020).

Abscisic acid (ABA) is a crucial phytohormone in plant defence mechanisms against drought stress. ABA plays a pivotal role in regulating physiological processes such as stomatal closure and stress‐responsive gene expression, which are essential for reducing water loss and improving plant survival under water‐deficient conditions (Yang et al. 2019). In response to drought stress, ABA is biosynthesised and perceived by its receptor complex, pyrabactin resistance 1 (PYR1)/PYR1‐like (PYL)/regulatory components of ABA receptors (RCAR), which inhibits 2C protein phosphatases (PP2Cs) (Ma et al. 2009; Park et al. 2009). This inhibition allows the activation of Snf1‐related protein kinase (SnRK2), which undergoes autophosphorylation and phosphorylates downstream targets, including transcription factors, to initiate plant defence responses (Cutler et al. 2010). Among the downstream targets of SnRK2 kinases, AREB/ABFs (ABA response element binding) are the primary transcription factors mediating ABA responses (Johnson et al. 2002; Kobayashi et al. 2005; Furihata et al. 2006; Fujii et al. 2007). Other transcription factors, including RAV and homeodomain‐leucine zipper (HD‐ZIP), have also been identified as key players (Feng et al. 2014; Tan et al. 2018; Baek et al. 2023).

The HD‐ZIP family is unique to plants and is classified into four subfamilies (I–IV) based on sequence conservation, structural features, and functional properties (Ariel et al. 2007; Agalou et al. 2008). HD‐ZIP proteins contain a conserved homeodomain (HD, comprising 61 amino acids) and an HB‐associated leucine zipper (HALZ, plant‐specific) element, which is tightly linked to the HD terminus. The HD is responsible for the specific DNA binding, and the leucine zipper mediates protein–protein interaction and DNA binding (Capella et al. 2016). Functional studies have extensively characterised numerous HD‐ZIP members, particularly those from subfamily I, in response to drought stress. However, HD‐ZIP subfamily II proteins have received comparatively less attention (Sharif et al. 2021; Li et al. 2022). For instance, in Arabidopsis, HD‐ZIP II protein HAT1 negatively regulates drought tolerance (Tan et al. 2018). In response to drought stress, HAT1 inhibits the expression of ABA biosynthesis genes *ABA3* and *NCED3* by binding their promoter and is phosphorylated by SnRK2.3, accelerating its degradation (Tan et al. 2018). Additionally, *Arabidopsis HAT22/ABIG1* expression is upregulated under drought conditions, and the increased expression levels, which mimic the effects of ABA application, are necessary for growth inhibition and leaf yellowing (Liu et al. 2016). In *Eucalyptus camaldulensis* , overexpression of the HD‐ZIP II gene *EcHB1* enhances drought tolerance by suppressing defoliation and improving tree growth (Sasaki et al. 2019). Various HD‐ZIP II genes also exhibit transcriptional alteration in response to drought stress. For example, the tomato homologue of *HAT22/ABIG1 SlABIG1* and four other HD‐ZIP II genes are differentially expressed in tomato leaves in response to drought stress (Ding et al. 2022). *OsHOX11* and *OsHOX27* transcripts from rice cultivars ( *Oryza sativa* ) dramatically decreased upon drought exposure, although *OsHOX27* was upregulated under mild drought stress (Agalou et al. 2008). In contrast, *OsHOX19* expression was upregulated under drought conditions in both drought‐sensitive and ‐resistant rice cultivars. The sesame ( *Sesamum indicum* ) HD‐ZIP II genes *SiHDZ03* and *SiHDZ42* are highly induced by drought stress, whereas *SiDHZ14* is downregulated (Wei et al. 2019). In the blue resurrection plant ( *Craterostigma plantagineum* ), *CpHB1* and *CpHB2* show different expression patterns; both transcripts are upregulated under water deficit, but in the presence of ABA, only *CpHB2* is further upregulated, while *CpHB1* shows no difference (Deng et al. 2002). Despite these findings, identification of the HD‐ZIP II genes involved in drought stress response remains limited, particularly in pepper plants ( *Capsicum annuum* ), and their molecular functions have yet to be fully elucidated.

In our previous study, the HD‐ZIP class II gene *CaHAT1* was identified as a positive regulator of drought tolerance in pepper plants (Baek et al. 2023). Under drought stress conditions, CaHAT1 is phosphorylated and activated by CaSnRK2.6 and positively regulates the expression of the drought stress‐responsive genes *CaOSR1* and *CaRAB18*, potentially by binding to their promoters. In this study, we aimed to identify additional transcription factors involved in drought stress defence in pepper, particularly those associated with the core ABA signalling pathway. We identified the HD‐ZIP II gene *CaDRHB12* (*C. annuum Drought Responsive Homeobox Gene 12*), whose expression decreases under ABA treatment or drought stress, contrasting with that of *CaHAT1*. Despite its high conservation with *CaHAT1*, *CaDRHB12* negatively regulates ABA signalling and drought stress responses. CaDRHB12 directly interacts with and is phosphorylated by CaSnRK2.6, causing the CaDRHB12 protein to become unstable. Our study suggests that the highly homologous genes *CaHAT1* and *CaDRHB12* are differentially regulated through phosphorylation by the same kinase, resulting in precise regulation of the complex responses to ABA and drought stress.

## 2 Materials and Methods

### 2.1 Plant Materials and Growth Conditions

Seeds of hot pepper ( *C. annuum* L. cultivar ‘Nockwang’) and tobacco (*Nicotiana benthamiana*) were sown and germinated in a compost soil mix (Sunshine Mix #5; Sun Gro Horticulture, Agawam, MA, USA) at 24°C. *Arabidopsis thaliana* (Col‐0) seeds were placed on a 0.8% agar MS plate with 1.5% sucrose and vernalized at 4°C in darkness for 2 days to ensure uniform germination. All plants were grown at 24°C with a 16/8 h light/dark photoperiod.

### 2.2 Virus‐Induced Gene Silencing

*CaDRHB12*‐silenced pepper plants were generated using the tobacco rattle virus (TRV)‐based virus‐induced gene silencing system as described previously (Lim et al. 2017). To reduce potential off‐target effects, a target region (1–300 bp) within CaDRHB12 (CA06g12600, *C. annuum* reference genome CM334 version 1.55) was predicted using the SGN virus‐induced gene silencing (VIGS) tool (http://vigs.solgenomics.net). The antisense fragment was subsequently inserted into the pTRV2 vector, producing the pTRV2‐*CaDRHB12* construct, which was introduced into *Agrobacterium tumefaciens* strain GV3101 using the freeze–thaw method. Subsequently, *Agrobacterium* cells carrying pTRV2:*CaDRHB12* were mixed with those containing pTRV1 (final OD_600_ = 0.2), and the mixture was co‐infiltrated into fully expanded cotyledons of pepper plants. A pTRV2:00 vector was used as a negative control.

### 2.3 Generation of Transgenic *Arabidopsis* Plants

*A. tumefaciens* ‐mediated transformation of *Arabidopsis* was performed using the floral dip method (Clough and Bent 1998). To generate *CaDRHB12*‐overexpressing lines, the coding sequence of *CaDRHB12* was cloned into the cauliflower mosaic virus 35S promoter‐driven green fluorescent protein (GFP) expression vector (p326GFP) using a gateway system (Invitrogen, Carlsbad, CA, USA). Floral dipping was conducted using the *A. tumefaciens* strain GV3101 containing a p326GFP‐*CaDRHB12* vector. Transgenic mutants were initially selected on MS agar medium containing 25 μg mL^−1^ phosphinothricin. The primers used for cloning and confirming transformation are listed in Table S1.

### 2.4 Subcellular Localization Analysis

To express GFP‐tagged CaDRHB12 in plants, the *A. tumefaciens* strain GV3101 carrying the p326GFP‐*CaDRHB12* vector was mixed with a p19 strain to avoid gene silencing (final OD_600_ = 0.5). Thereafter, the mixture was infiltrated into the abaxial side of tobacco leaves using a 1‐mL needleless syringe. After 2 days of agroinfiltration, GFP signals were observed using confocal microscopy (Zeiss 710 UV/Vis Meta; Carl Zeiss AG, Oberkochen, Germany) operated with LSM Image Browser software (Carl Zeiss AG).

### 2.5 RNA Isolation and RT‐qPCR

Total RNA was isolated from the leaves of hot pepper and *Arabidopsis* plants subjected to drought or ABA (100 μM) treatment using an RNeasy Mini kit (Qiagen, Valencia, CA, USA). From all RNA samples (1 μg), cDNA was synthesised using a Transcript First Strand cDNA Synthesis Kit (Roche Diagnostics Corporation, Indianapolis, IN, USA), following the manufacturer's protocols. RT‐qPCR was conducted using iQ SYBR Green Supermix (Bio‐Rad Laboratories, Hercules, CA, USA) with appropriate primers (Table S1) on a CFX96 Touch Real‐time PCR detection system (Bio‐Rad Laboratories). For normalisation, *Actin1* (*CaACT1*) from pepper and *Actin8* (*AtACT8*) from *Arabidopsis* genes were used as internal controls. Each reaction was performed in triplicate using three independent experiments.

### 2.6 Transactivation Analysis in Yeast Cells

Transactivation of CaDRHB12 protein in yeast cells was analysed as previously described (Lim et al. 2021). Briefly, the *CaDRHB12* gene sequence was divided into fragments corresponding to its distinct functional domains. The full‐length *CaDRHB12* gene and its fragments were cloned into a pGBKT7 vector (Clontech Laboratories, Mountain View, CA, USA) to fuse with the GAL4 DNA‐binding domain. Using the lithium acetate transformation method, these vectors, along with the pGADT7 empty vector, were transformed into yeast strain Y2H Gold. Subsequently, the transformed yeast cells were grown on selective SD minimal medium (Clontech Laboratories).

### 2.7 Recombinant Protein Production

The recombinant protein was produced and purified using glutathione‐S‐transferase (GST) (Thermo Fisher Scientific, Waltham, MA, USA) and maltose‐binding protein (MBP) (New England Biolabs, Ipswich, MA, USA) according to the manufacturer's instructions. Briefly, MBP‐tagged CaDRHB12 protein was expressed in *Escherichia coli* cells through treatment with 1 mM isopropyl‐b‐d‐thiogalactoside (IPTG) and culturing for 3 h. The soluble fraction of the bacterial lysate was collected through centrifugation and sonication. Following incubation with amylose resin (New England Biolabs) for 3 h at 4°C, the resin sample was washed with 1 mL washing buffer (binding buffer containing 0.5% Triton X‐100). Thereafter, the recombinant proteins were eluted by adding a 10 mM maltose solution. Similarly, GST‐tagged CaSnRK2.6 was purified using a Fast‐4‐flow glutathione resin (GE Healthcare, Chicago, IL, USA), and elution was conducted using an elution buffer containing reduced glutathione.

### 2.8 Pull‐Down Assay

A pull‐down assay was conducted as previously described (Lim et al. 2021). Briefly, amylose resin‐bound MBP‐CaDRHB12 protein was mixed and incubated with pre‐cleared GST‐CaSnRK2.6 and GST at 4°C for 2 h. Following washing five times with a washing buffer (20 mM Tris‐HCl, 150 mM NaCl, 5 mM EDTA, and 0.5% Triton X‐100), the samples were eluted by adding a GST‐elution buffer (20 mM Tris‐HCl, 150 mM NaCl, and reduced glutathione) and then mixed with a 4× Laemmli protein buffer. Western blotting was conducted using GST (Santa Cruz Biotechnology, Dallas, TX, USA) and MBP (New England Biolabs) antibodies. The GST protein was used as a negative control.

### 2.9 Bimolecular Fluorescence Complementation (BiFC) and Split‐Luciferase Complementation (SLC) Assay

For the BiFC assay, *CaDRHB12* and *CaSnRK2.6* cDNA were cloned into VYNE and CYCE vectors, respectively, which harbour the N‐ and C‐terminal fragments of yellow fluorescent protein. *A. tumefaciens* strain GV3101 harbouring these constructs was mixed with a p19 strain (final OD_600_ = 0.5, 1:1:1 ratio), and the mixture was infiltrated into the abaxial side of *N. benthamiana* leaves using a 1 mL needleless syringe. The fluorescence signals were observed 3 days after agroinfiltration using confocal microscopy, as described previously.

The SLC assay was performed according to a previous report with some modifications (Gehl et al. 2011). Briefly, the full‐length cDNA of CaDRHB12 and CaSnRK2.6 was cloned into pDEST14‐N‐LUC (containing an N‐terminal luciferase fragment) and pDEST14‐C‐LUC (fused with a C‐terminal luciferase fragment) vectors, respectively. Agroinfiltration was conducted as described above for the BiFC assay. After 3 days, the leaf samples were infiltrated with 0.1 mM d‐luciferin (BioVision, Milpitas, CA, USA) and further incubated for 30 min in the dark. Luminescence signals were observed using a NightShade evo LB 985N In Vivo Plant Imaging System (Berthold Technologies GmbH & Co. KG, Bad Wildbad, Germany). Image processing and luminescence quantification were performed using IndiGO software (version 3.0; Berthold Technologies GmbH & Co. KG).

### 2.10 Co‐Immunoprecipitation Assay and Protein Degradation *in Planta* Analysis

Using agroinfiltration, GFP‐tagged CaSnRK2.6 and 3×FLAG‐tagged CaDRHB12 were transiently co‐expressed in *N. benthamiana* leaves. After 3 days of agroinfiltration, the leaves were infiltrated with 50 μM MG132 and harvested after 12 h. Afterward, total leaf protein was extracted using a GTEN extraction buffer [5 mM DTT, 10% glycerol, 0.5 M sucrose, 150 mM Tris‐MES (pH 7.5), 1 mM EDTA, 150 mM NaCl, complete protease inhibitor (Roche), 0.2% Triton X‐100, and 2% PVPP]. Next, the extracted protein samples were incubated with anti‐GFP magnetic agarose (ChromoTek GmbH, Planegg‐Martinsried, Germany), followed by an immunoblot assay performed using anti‐GFP and anti‐FLAG antibodies. This method was also used to analyse *in planta* CaDRHB12 protein degradation in the presence of CaSnRK2.6 or CaSnRK2.6^K52N^.

### 2.11 Cell‐Free Degradation Assay

Crude extracts were prepared from the leaves of *CaSnRK2.6*‐silenced pepper plants and *CaSnRK2.6*‐overexpressing (OX) transgenic *Arabidopsis* plants using cell‐free extraction buffer [1 mM ATP, 10 mM MgCl_2_, 10 mM NaCl, 25 mM Tris‐HCl (pH 7.5), 5 mM DTT, and 0.1% triton X‐100]. Thereafter, the crude extract samples (50 μg) were incubated with MBP‐tagged CaDRHB12 and its mutant proteins at the indicated time points in the absence and presence of 50 μM MG132. Next, immunoblot analyses were conducted using an anti‐MBP antibody (New England Biolabs). The large rubisco subunit was used as a loading control and was visualised through Coomassie blue staining in SDS‐PAGE gels.

### 2.12 Dual‐Luciferase Reporter Assay

A dual‐luciferase assay was conducted, as described previously (Lim et al. 2021). The coding sequences of CaDRHB12, CaDRHB12^S15AS142A^, and CaSnRK2.6 were cloned into a 35S promoter‐driven pGWB2 vector as the effector. The reporter was generated by fusing the promoters of the drought‐responsive genes *CaRAB18* and *CaOSR1* (Lim, Baek, and Lee 2018) to the N‐terminus of the firefly luciferase (*FLuc*) gene in the pGWB35 vector. The Renilla luciferase (*RLuc*) gene, driven by the 35S promoter, was used as the internal control. Afterward, these constructs were transformed into *A. tumefaciens* strain GV3101, and a combination of effector, receptor, and internal control constructs was transiently co‐expressed into *N. benthamiana* leaves via agroinfiltration. After 3 days, luciferase activity was measured using an ELISA reader (SYNERGY HTX; BioTek Instruments, Winooski, VT, USA) and a Dual‐luciferase Reporter Assay System kit (Promega, Madison, WI, USA).

### 2.13 Electrophoretic Mobility Shift Assay

For electrophoretic mobility shift assay (EMSA), the MBP‐tagged CaDRHB12 protein was expressed in *E. coli* (BL21 DE3) by induction with 0.1 mM IPTG and purified using amylose resin according to the manufacturer's instructions (NEB). The probe, containing the ATHB6COREAT (CAATTATTA) sequence at position −1564, was synthesised and biotin‐labelled by Macrogen Co. After annealing to form a double‐stranded probe, EMSA was performed using the LightShift Chemiluminescent EMSA Kit (Thermo Fisher). An unlabeled probe (cold probe) was used as a competitor.

### 2.14 Drought Tolerance Assay

Drought stress responses in mature plants were observed by subjecting pepper plants at the four‐leaf stage and 3‐week‐old *Arabidopsis* plants to water deprivation under the aforementioned growth conditions. Following drought treatment, plants were rehydrated by watering for 3 days, and their recovery responses were evaluated.

To examine leaf transpirational water loss, the rosette leaves of 3‐week‐old *Arabidopsis* plants and the 1st and 2nd leaves of pepper plants at the four‐leaf stage were detached and subjected to gradual dehydration under controlled room conditions. The fresh weight of the detached leaves was recorded at the indicated time points to monitor water loss.

For thermal imaging analysis, 4‐week‐old pepper plants with fully expanded first and second leaves and 3‐week‐old *Arabidopsis* plants were sprayed with 100 μM ABA. Subsequently, thermal images were obtained using an infrared camera (FLIR T420; Teledyne FLIR, Wilsonville, OR, USA), and leaf temperatures were measured using FLIR Tools+ ver 5.2 software (Teledyne FLIR). For stomatal aperture measurements, epidermal peels were stripped from pepper and *Arabidopsis* leaves and floated in a stomatal opening solution [SOS: 50 mM KCl, 10 mM MES‐KOH (pH 6.5), and 10 μM CaCl_2_] under light conditions (Cheong et al. 2007). After incubation for 3 h, the buffer was replaced with fresh SOS containing 0, 10 and 20 μM ABA (Sigma‐Aldrich, St. Louis, MO, USA). After additional incubation for 2.5 h, 100 stomata were randomly selected, and their pore sizes were measured in each sample.

## 3 Results

### 3.1 Molecular Characterisation of CaDRHB12

In our previous study, *C. annuum* homeobox ABA signalling‐related transcription factor 1 (*CaHAT1*), an interacting partner of CaSnRK2.6, was found to play a positive role in drought response, and its activity was enhanced through phosphorylation by CaSnRK2.6 (Baek et al. 2023). Here, we isolated a different homeobox gene, *CaDRHB12* (CA06g12600; Lim, Hong, et al. 2018), which shares 66% identity and 72.9% similarity in the amino acid sequence with CaHAT1 in the pepper genome (Figure S1). CaDRHB12 exhibits the characteristic domain architecture of the HD‐ZIP II family, comprising an HD‐ZIP N‐terminal, a homeobox (HOX) and a HALZ. In the N‐terminal region, CaDRHB12 contains an EAR motif (LGLSL; residues 8 to 12), potentially implicated in transcriptional repressor activity (Brandt et al. 2014) and interaction with co‐repressor (Causier et al. 2011), and a CPSCE motif (residues 258 to 264), responsible for the DNA‐binding activity of CaHAT1 (Baek et al. 2023). Based on the structural genetic similarity, we hypothesised that *CaDRHB12* is homologous to *CaHAT1*.

**FIGURE 1:**
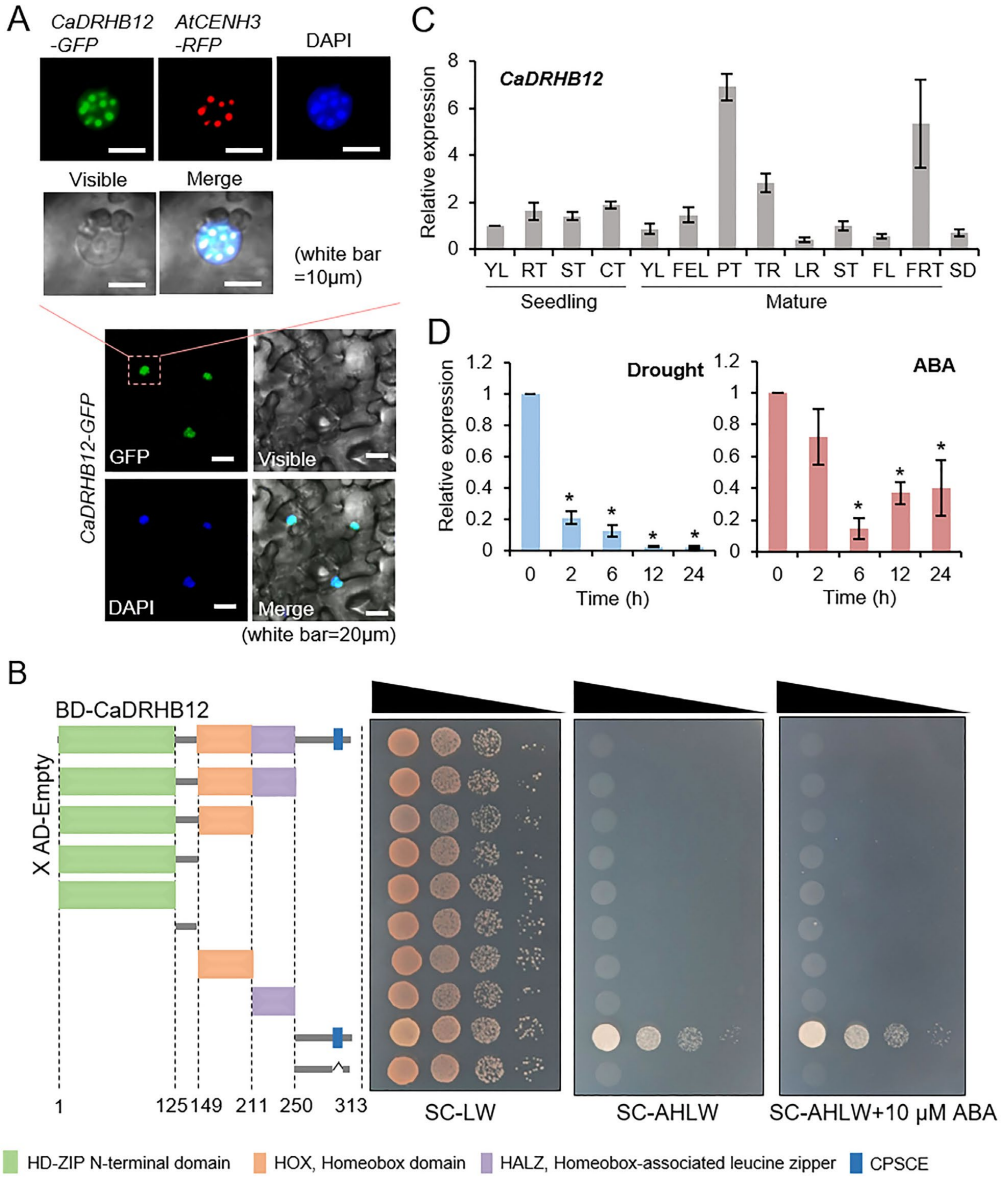
Molecular characterisation of CaDRHB12. (A) Subcellular localization of GFP‐tagged CaDRHB12 protein in leaf epidermal cells of *Nicotiana benthamiana* plants. *Pro35S:CaDRHB12‐GFP* was transiently expressed in *N. benthamiana* leaves through agroinfiltration, and GFP signals were observed after 2 days using confocal microscopy. The blue fluorescence signal corresponds to the nuclear marker 4′, 6‐diamidino‐2‐phenylindole (DAPI). AtCENH3, fused with red fluorescent protein, served as a centromere marker. (B) DNA‐binding activity of CaDRHB12. Domain analyses were performed using the Simple Modular Architecture Research Tool program, which produced the schematic representation shown in the left panel. CaDRHB12 was truncated based on its functional domain, cloned into a pGBKT7 vector, and transformed into Gold‐strain yeast cells. Transformed yeast cells were grown on control (SC‐Leu/‐Trp, LW) and selection (SD/‐Ade‐His‐Leu‐Trp, AHLW) media in the presence or absence of 10 μM ABA. (C) Organ‐specific expression patterns of *CaDRHB12* in pepper seedlings and mature plants. CT, cotyledon; FEL, fully expanded leaf; FL, flower; FRT, fruit; LR, lateral root; PT, petiole; RT, root; SD, seed; ST, stem; TR, tap root; YL, young leaf. (D) *CaDRHB12* expression levels in pepper plants subjected to dehydration by leaf detachment (left) and ABA treatment (100 μM, right). The pepper *Actin1* (*CaACT1*) was used as an internal control. All data (ΔΔCT values) represent the mean of three independent experiments (with > 95% confidence). Asterisks denote statistically significant differences compared with the control sample (Student's *t*‐test; *p < 0.05).

Confocal microscopic analysis revealed that GFP‐tagged CaDRHB12 protein localised predominantly within the nucleus in the leaf epidermal cells of *N. benthamiana* (Figure 1A). Notably, the GFP signals overlapped with the red fluorescence emitted by RFP‐tagged AtCENH3, encoding a centromere‐specific histone H3. Similar to CaHAT1, CaDRHB12 exhibits DNA‐binding activity, as demonstrated by the GAL4 yeast system (Figure 1B). Upon co‐transforming the AD‐empty vector with CaDRHB12 fragments cloned into the pGBKT7 vector into yeast, we observed that only the yeast cells containing the C‐terminal fragments (residues 250–313) were able to grow on the selection medium. To test whether this transactivation activity depends on the conserved CPSCE motif, we generated a deletion variant, CaDRHB12 (250–313)ΔCPSCE, lacking these conserved residues. Unlike the CaDRHB12 (250–313) fragment, CaDRHB12 (250–313)ΔCPSCE variant failed to support yeast growth on the selection medium (Figure 1B). These results suggest that the C‐terminal region, particularly the CPSCE motif, is essential for the transactivation activity of CaDRHB12.

Next, we analysed the *CaDRHB12* expression patterns in various pepper organs (Figure 1C). RT‐qPCR analysis revealed that *CaDRHB12* expression levels were higher in petioles and fruits. We further investigated how *CaDRHB12* expression is altered in response to drought stress and ABA treatment in pepper leaves (Figure 1D). Drought stress resulted in gradual downregulation of *CaDRHB12* expression, with an 80% reduction observed within 2 h after treatment compared with that in healthy leaves. Similarly, *CaDRHB12* levels decreased by over 80% 6 h post‐ABA treatment.

### 3.2 Silencing of *CaDRHB12* Expression Leads to Elevated Drought Stress Resistance

**FIGURE 2:**
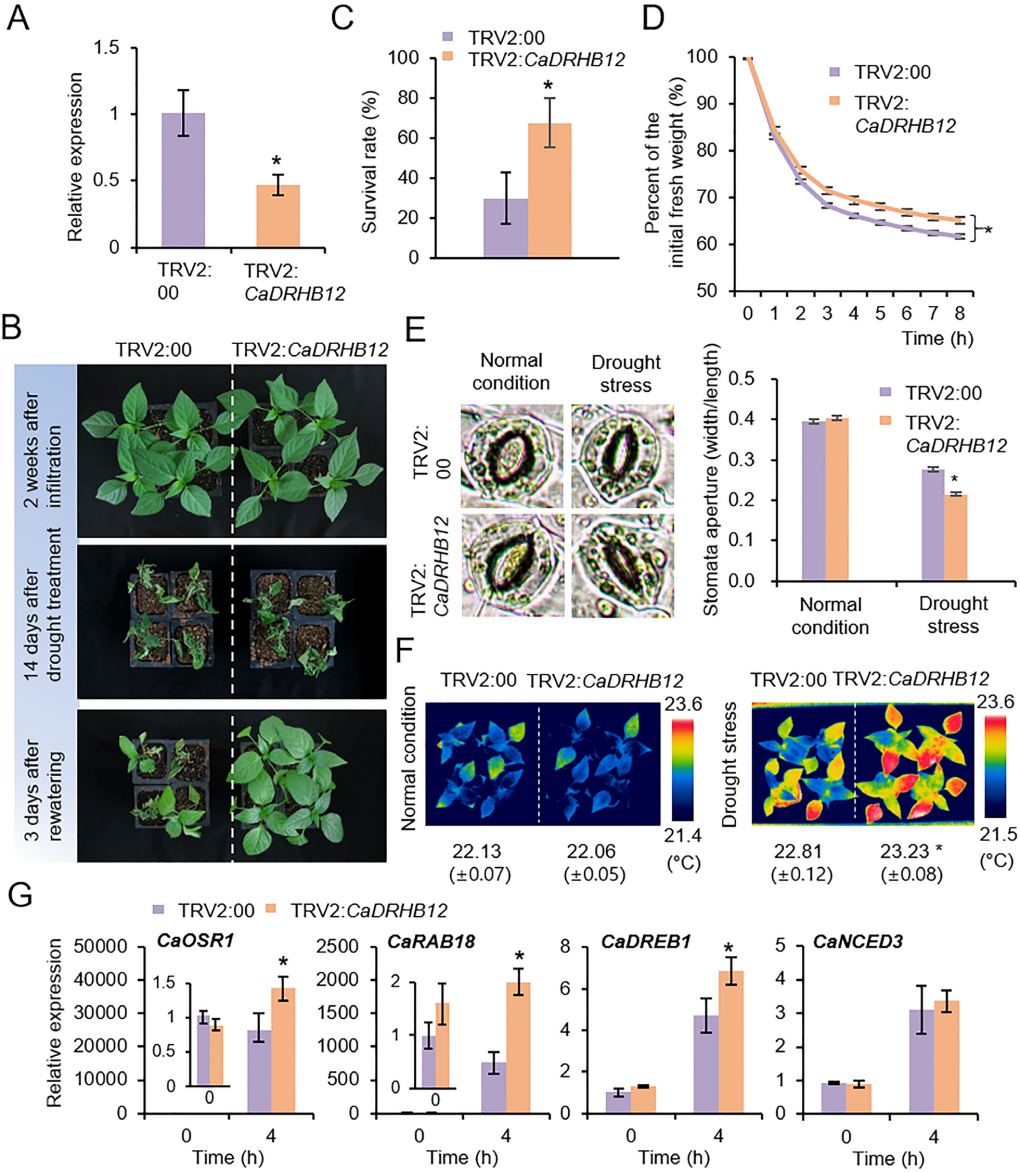
Drought sensitivity analysis of *CaDRHB12*‐silenced pepper plants. (A) *CaDRHB12* expression levels. RT‐qPCR analysis was conducted to evaluate the *CaDRHB12* transcript levels in control (TRV2:00) and *CaDRHB12*‐silenced (TRV2:*CaDRHB12*) pepper plants. (B, C) Enhanced drought tolerance of TRV2:*CaDRHB12* pepper plants. TRV2:00 and TRV2:*CaDRHB12* pepper plants grown for 2 weeks post‐agroinfiltration were exposed to drought stress by growing them without water for 14 days. Representative images were obtained before (top) and after (middle) drought treatment (B). At 3 days after rewatering (bottom), the survival rates were evaluated by counting the number of surviving plants (C). (D) Transpirational water loss from the leaves of TRV2:00 and TRV2:*CaDRHB12* pepper plants. The fresh weights of leaf samples were recorded 8 h after leaf detachment (*n* = 16 plants). (E) Stomatal apertures of TRV2:00 and TRV2:*CaDRHB12* pepper plants in response to drought stress. Each pepper plant (4‐week‐old) were subjected to drought stress (or well‐watered treatment) for 7 days, then leaf peels were harvested. Representative images were taken (left) and the stomatal apertures were measured using Image J program (right). (F) Comparison in leaf surface temperature between control and TRV2:*CaDRHB12* pepper plants in response to drought stress. Following the above treatment of (E), the leaves from each line (*n* = 20) were observed and captured by thermal imaging camera. The leaf surface temperatures of each plant were measured using FLIR program. (G) Differential expression of drought‐responsive marker genes in the leaves of TRV2:00 and TRV2:*CaDRHB12* plants after drought treatment measured 0 and 4 h after detachment. The expression levels of each gene were normalised to that of *CaACT1*, with the expression in TRV2:00 at 0 h serving as the baseline (set to 1.0). All data represent the means ± SD of three independent experiments. Asterisks indicate significant differences between TRV2:00 and TRV2:*CaDRHB12* pepper plants (Student's *t*‐test; *p < 0.05).

To specify the function of CaDRHB12, we first performed phenotypic analyses using *CaDRHB12*‐silenced pepper plants under drought and ABA treatment conditions (Figure 2). The VIGS technique was applied to transiently knock down *CaDRHB12* in pepper plants. RT‐qPCR analysis showed that *CaDRHB12* gene expression was significantly lower in TRV2:*CaDRHB12* pepper leaves than in TRV2:00 pepper leaves (Figure 2A). After 2 weeks of agroinfiltration, these two plant lines were subjected to drought stress by withholding water for 14 days and rewatering for 3 days (Figure 2B). TRV2:*CaDRHB12* pepper showed drought tolerance phenotypes compared with TRV2:00 plants. Upon rewatering for 3 days, 67.34% of TRV2:*CaDRHB12* pepper plants survived against only 29.94% of the control pepper plants (Figure 2C).

We considered whether the enhanced drought tolerance of TRV2:*CaDRHB12* plants is attributed to the modulation of transpiration through stomatal pores in the leaves. To test this, we determined transpirational water loss from the leaves of TRV2:*CaDRHB12* and TRV2:00 pepper plants (Figure 2D). After detaching the leaves and maintaining the same conditions for 8 h, we found that TRV2:*CaDRHB12* leaves retained significantly higher fresh weights than those of TRV2:00 leaves. Additionally, we assessed stomatal apertures in the leaves of the two plant lines after withholding water for 7 days (Figure 2E). Under normal growth conditions, the stomatal apertures in *CaDRHB12*‐silenced plants were comparable to those in control plants. However, under drought stress conditions, both pepper plants exhibited stomatal closure and, notably, TRV2:*CaDRHB12* pepper plants displayed more pronounced closure than TRV2:00 plants. Parallel thermal imaging analysis revealed that dehydrated leaves of TRV2:*CaDRHB12* exhibited higher surface temperatures compared to those of TRV2:00 leaves (Figure 2F). Consistent with drought‐induced ABA accumulation, exogenous application of ABA similarly elicited enhanced stomatal closure and elevated leaf temperatures in TRV2:*CaDRHB12* plants (Figure S2). Collectively, the results showed that compared with that of control plants, *CaDRHB12*‐silenced pepper plants showed improved drought tolerance associated with leaf transpiration regulation.

Next, we examined the expression levels of pepper drought stress/ABA‐responsive marker genes in the leaves of TRV2:*CaDRHB12* and TRV2:00 pepper plants subjected to drought stress (Figure 2G). RT‐qPCR analysis revealed that *CaOSR1*, *CaRAB18* and *CaDREB1* expression levels were significantly upregulated in TRV2:*CaDRHB12* pepper compared with those in TRV2:00 pepper. In contrast, *CaNCED3* expression between TRV2:*CaDRHB12* and TRV2:00 pepper plants did not significantly differ. These findings suggest that CaDRHB12 negatively contributes to drought response by modulating the stomatal aperture and stress‐responsive genes.

### 3.3 Overexpression of *CaDRHB12* Decreases Plant Resistance to Drought Stress in Arabidopsis Plants

**FIGURE 3:**
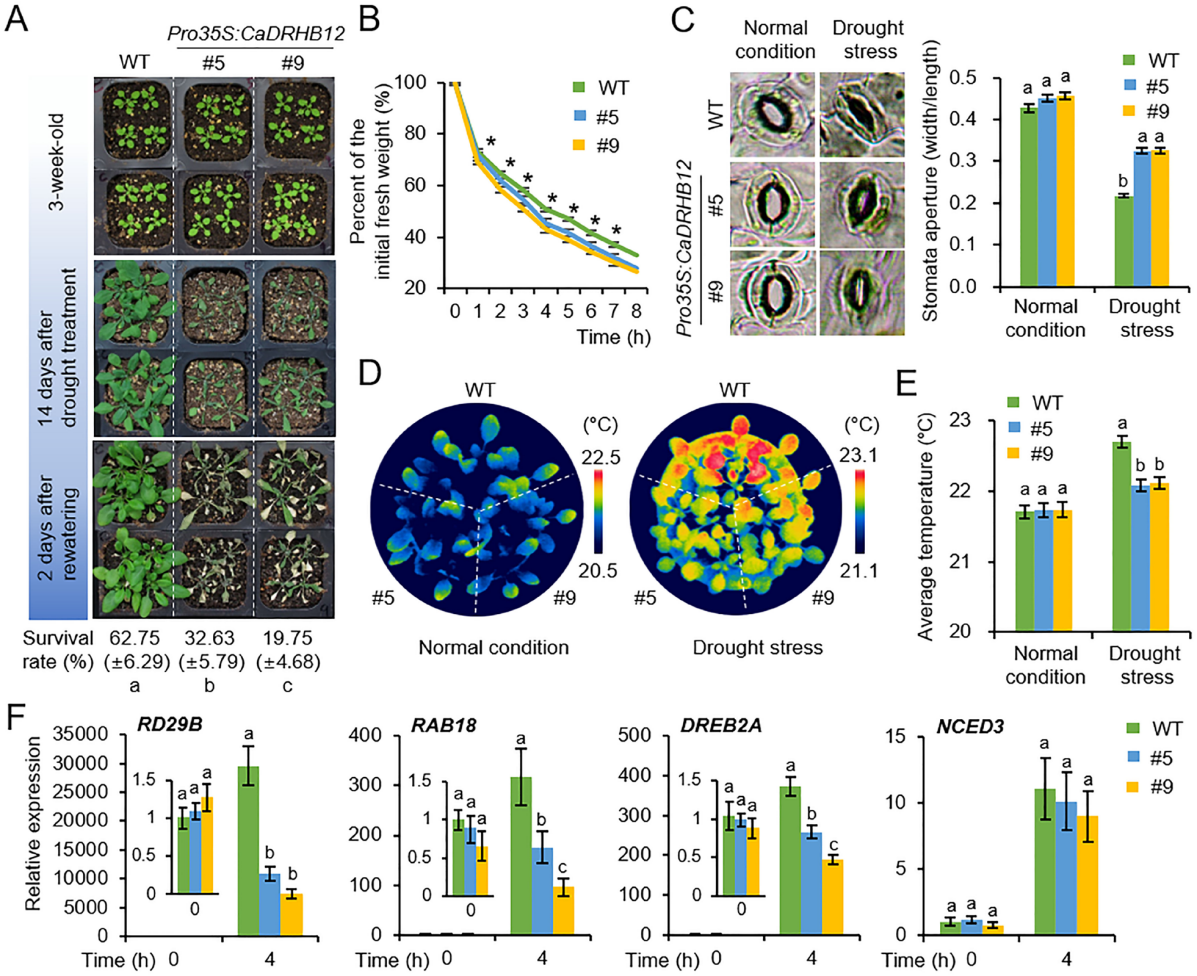
Drought sensitivity analysis of *CaDRHB12*‐overexpressing *Arabidopsis* plants. (A) Reduced drought tolerance in *Pro35S:CaDRHB12* plants. Three‐week‐old wild‐type (WT) and *Pro35S:CaDRHB12* plants were exposed to drought stress by growing them without water for 14 days. Representative images were obtained before (top) and after (middle) drought treatment. At 3 days after rewatering (bottom), the survival rates were evaluated by counting the number of surviving plants. (B) Transpirational water loss from the rosette leaves of WT and *Pro35S:CaDRHB12* pepper plants. The fresh weights of leaf samples were recorded 8 h after leaf detachment (*n* = 16 plants). (C, D) Leaf temperatures of WT and *Pro35S:CaDRHB12* plants with or without drought stress. Representative thermographic images were taken at 10 days after ± drought treatment (C), and the temperatures were measured by the thermal imaging camera and calculated from the three largest leaves of plants from each line (*n* = 12) (D). (E) Drought stress‐induced stomatal closure in WT and *Pro35S:CaDRHB12* plants. Stomatal apertures were measured 10 days after ± drought treatment. Representative images of the stomata were obtained for the leaves of each line (left) and the apertures of 100 randomly selected stomata were measured (right). (F) Quantitative RT‐PCR analysis of ABA‐responsive genes in wild‐type and *CaDRHB12* overexpressing plants after treatment with ABA. All data represent the mean ± SD of three independent experiments.

To further investigate the functional role of CaDRHB12 in drought stress response, we utilised *A. thaliana* , a model plant amenable to *Agrobacterium*‐mediated stable gene transformation, unlike pepper plants (Figure 3). Two independent lines (T3) of *Pro35S:CaDRHB12 Arabidopsis* plants exhibiting high *CaDRHB12* expression were selected for further study (Figure S3A,B). Drought tolerance was evaluated by subjecting these transgenic lines and wild‐type plants to a 14‐day period of water deprivation and rewatering (Figure 3A). Under normal conditions, no discernible phenotypic differences were observed between *Pro35S:CaDRHB12* and wild‐type plants (Figure 3A, top panel). However, under drought stress conditions, *Pro35S:CaDRHB12* plants exhibited significant wilting compared with that of wild‐type plants (Figure 3A, middle panel). Following rewatering for 2 days, the survival rate of wild‐type plants was 62.75%, while those of the *Pro35S:CaDRHB12* lines were 32.68% and 19.75% (Figure 3A, bottom panel). The drought‐sensitive phenotype of *Pro35S:CaDRHB12* lines was hypothesised to be associated with the modulation of transpirational water loss through leaf stomata. To test this, we monitored the leaf transpiration rate by measuring the fresh weights of detached leaves hourly for 8 h (Figure 3B). Compared with that of wild‐type plants, the *Pro35S:CaDRHB12* plant lines exhibited significantly reduced leaf water content. Furthermore, we measured the stomatal pore sizes of *Pro35S:CaDRHB12* and wild‐type plants following withholding water for 10 days (Figure 3C). Under normal growth conditions, the stomatal apertures in *Pro35S:CaDRHB12* plants were comparable to those in wild‐type plants. However, under drought stress conditions, both plant lines exhibited stomatal closure and notably, the stomatal pore sizes of *Pro35S:CaDRHB12* were significantly smaller than those of wild‐type plants. Thermal imaging further demonstrated that dehydrated leaves of *Pro35S:CaDRHB12* showed lower surface temperatures compared to those of wild‐type leaves (Figure 3D,E). Similar patterns in stomatal aperture and leaf temperature were also observed following ABA treatment (Figure S3C,D). We also investigated the expression levels of *Arabidopsis* drought stress/ABA‐responsive marker genes, including *RD29B*, *RAB18*, *DREB2A*, and *NCED3*, in both wild‐type and *Pro35S:CaDRHB12* lines (Figure 3F). RT‐qPCR analysis showed that the expression levels of all marker genes, except *NCED3*, were significantly lower in *Pro35S:CaDRHB12* than in wild‐type plants after drought stress treatment. These results suggest that CaDRHB12 negatively regulates drought tolerance in *Arabidopsis*, similar to its observed effects in pepper plants.

### 3.4 CaDRHB12 Interacts With CaSnRK2.6

**FIGURE 4:**
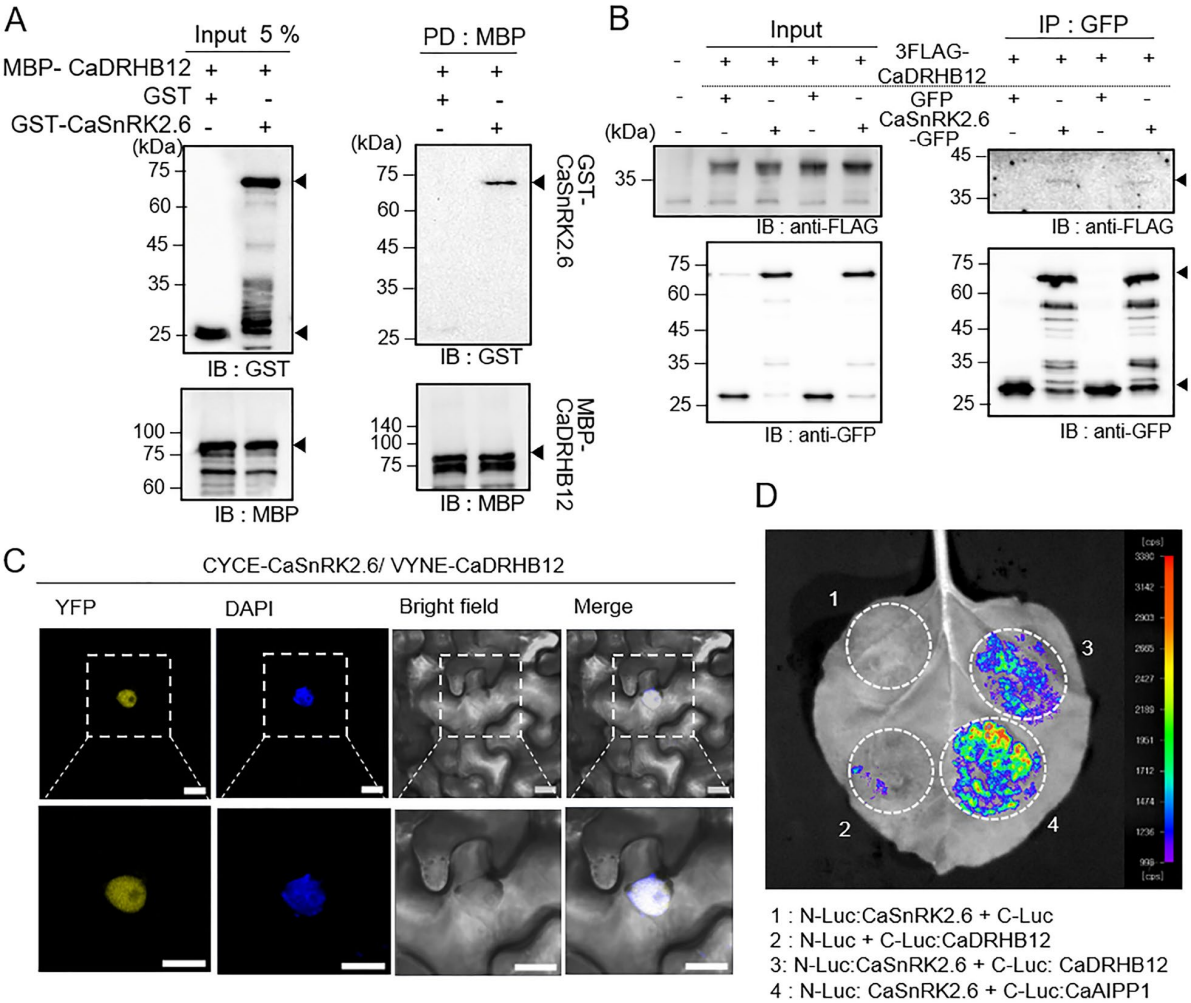
Physical interaction of CaDRHB12 with CaSnRK2.6. (A) In vitro pull‐down assay of MBP‐CaDRHB12 and GST‐CaSnRK2.6. MBP‐CaDRHB12 proteins bound to amylose resin were incubated with the total lysate of GST‐CaSnRK2.6 or GST (as a negative control), and immunoblot analysis was conducted using anti‐MBP and anti‐GST antibodies. (B) Co‐immunoprecipitation analysis. Through agroinfiltration, 3×FLAG‐CaDRHB12 was transiently co‐expressed with GFP (as a negative control) or CaSnRK2.6‐GFP in the leaves of *Nicotiana benthamiana* plants. After 3 days, total leaf extracts were precipitated using anti‐GFP magnetic beads and subjected to immunoblot analysis using anti‐FLAG and anti‐GFP antibodies. The input (left panel) contains 10% of the total leaf extracts. Black arrowheads indicate the tagged target proteins. (C) Bimolecular fluorescence complementation analysis. VYNE:CaDRHB12 and CYCE:CaSnRK2.6 were co‐expressed in the *N. benthamiana* leaves through agroinfiltration, followed by confocal microscopy observation. DAPI (blue signal) was used as a nuclear indicator. White bar = 20 μm. (D) Split‐luciferase complementation analysis. C‐LUC:CaDRHB12 and N‐LUC:CaSnRK2.6 were co‐expressed in *N*. *benthamiana* as described above. After 3 days, a solution of 0.1 M d‐luciferin was infiltrated, and their interaction was observed using a CCD camera. The empty vector was used as a negative control, and the interaction between CaSnRK2.6 and CaAIPP1 was used as a positive control.

Our recent findings revealed that the ABA signalling‐related SnRK2 kinase CaSnRK2.6 phosphorylates and activates CaHAT1, thereby enhancing its downstream gene expression and subsequently increasing drought tolerance (Baek et al. 2023). Given the high sequence homology between CaHAT1 and CaDRHB12, we investigated potential interactions between CaDRHB12 and CaSnRK2.6 (Figure 4). To this end, a pull‐down assay was conducted using recombinant protein MBP‐tagged CaDRHB12 and GST‐tagged CaSnRK2.6. After the pull‐down of the MBP‐tagged protein using amylose resin, immunoblot analysis with anti‐GST showed that CaDRHB12 interacted specifically with CaSnRK2.6 and not with the GST protein alone (Figure 4A). The physical interaction between CaDRHB12 and CaSnRK2.6 was also observed *in planta* (Figure 4B–D). Co‐immunoprecipitation using anti‐GFP magnetic beads followed by immunoblot analysis with an anti‐FLAG antibody confirmed the interaction between CaDRHB12 and CaSnRK2.6 (Figure 4B). This interaction *in planta* was further supported by data from BiFC (Figure 4C) and SLC assays (Figure 4D). Notably, CaDRHB12 interacted with CaSnRK2.6 in the nucleus (Figure 4C), with no fluorescence signals observed in the empty vector control (Figure S4). Our findings suggest that CaDRHB12 could be a potential substrate for CaSnRK2.6.

### 3.5 ABA Signalling‐Related Kinase CaSnRK2.6 Directly Phosphorylates CaDRHB12

**FIGURE 5:**
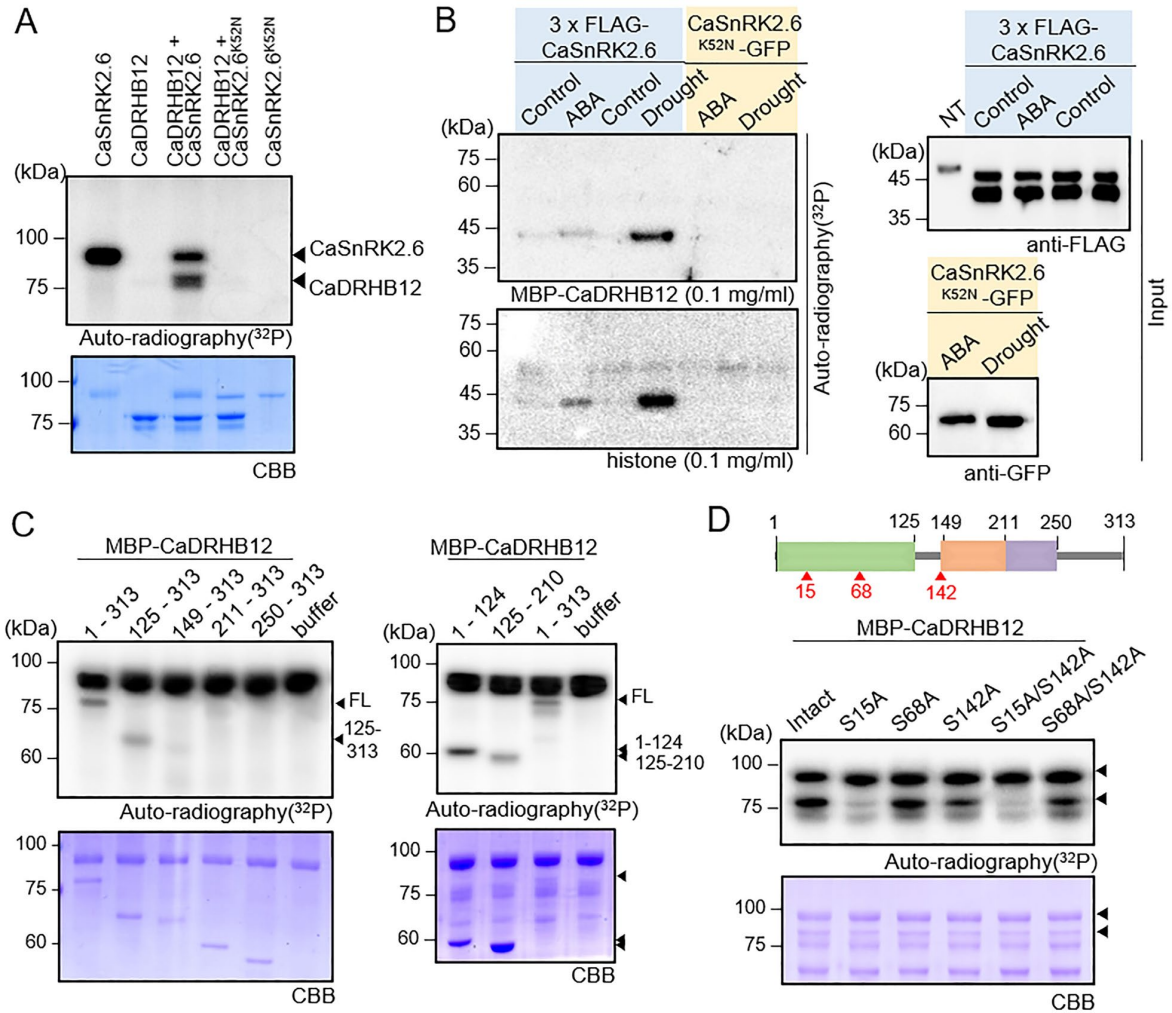
CaSnRK2.6‐mediated phosphorylation of CaDRHB12. (A) Phosphorylation of CaDRHB12 depends on the kinase activity of CaSnRK2.6. For the in vitro kinase assay, an equal amount of purified CaDRHB12 proteins was incubated with CaSnRK2.6 or its kinase‐dead mutant CaSnRK2.6^K52N^. Radio‐labelled signals were visualised using phosphoscreen detection. (B) CaSnRK2.6‐mediated phosphorylation of CaDRHB12 in response to ABA and drought stress. Through agroinfiltration, 3×FLAG‐CaSnRK2.6 or CaSnRK2.6^K52N^‐GFP was transiently expressed in *Nicotiana benthamiana* leaves, and after 2 days, the infiltrated leaves were treated with 100 μM ABA for 2 h or detached for 30 min. For the in‐gel kinase assay, 2 μg plant extract protein was loaded onto SDS‐PAGE gel containing CaDRHB12 protein or histone as a Ser/Thr kinase substrate. The input (right panel) displays the CaSnRK2.6 and CaSnRK2.6^K52N^ protein expression levels based on immunoblot analysis. (C) Phosphorylation of the N‐terminal region of CaDRHB12 by CaSnRK2.6. Fragmented CaDRHB12 proteins, based on their functional domains, were incubated with an equal amount of CaSnRK2.6 protein. Coomassie brilliant blue (CBB) staining (below) indicated protein input in the kinase assay. (D) Detection of serine residues in CaDRHB12 for CaSnRK2.6‐mediated phosphorylation. Phosphorylation sites on CaDRHB12 for Ser/Thr kinase were predicted using the MusiteDeep web tool (https://www.musite.net/). For the in vitro kinase assay, CaDRHB12 and its variants, in which serine 15, serine 68, and/or serine 142 were substituted with alanine (bottom arrowhead), were incubated with CaSnRK2.6 (upper arrowhead). Coomassie brilliant blue (CBB) staining (below) indicated protein input in the kinase assay.

Based on the interaction between CaDRHB12 and CaSnRK2.6, we conducted kinase assays to ascertain whether CaDRHB12 is a direct phosphorylation target of CaSnRK2.6 (Figure 5). As shown in Figure 5A, CaDRHB12 was phosphorylated by active kinase CaSnRK2.6, whereas no phosphorylation occurred with the inactive kinase mutant CaSnRK2.6^K52N^. Since CaSnRK2.6 kinase is activated by drought stress (Jeong et al. 2023), we examined the effect of drought stress and ABA treatment on the CaSnRK2.6‐mediated phosphorylation of CaDRHB12 protein (Figure 5B). For the in‐gel kinase assay, *N. benthamiana* plants transiently co‐expressing *CaDRHB12* and *CaSnRK2.6* were subjected to drought stress and sprayed with 100 μM ABA solution after 2 days of agroinfiltration, and leaf total proteins were extracted. As expected, histone, used as a substrate for Ser/Thr protein kinases, was strongly phosphorylated by CaSnRK2.6 kinase in response to drought and ABA treatment relative to normal conditions. Correspondingly, CaDRHB12 phosphorylation was dependent on CaSnRK2.6 kinase activity and was not induced by CaSnRK2.6^K52N^, indicating that drought and ABA promote CaDRHB12 phosphorylation mediated by CaSnRK2.6.

Next, to identify the phosphorylation sites on CaDRHB12, we fragmented the protein according to its domains (Figure 5C). In vitro kinase assays revealed phosphorylation in the full‐length CaDRHB12 (1–313) and 125–210 amino acid fragments containing the homeobox domain. Moreover, the strongest phosphorylation signal was observed in the N‐terminal 1–124 region. To predict potential phosphorylation sites within this region, we used the Musite web tool (https://musite.sourceforge.net/), which identified three serine residues as candidates: Ser15, Ser68 and Ser142. These serine residues were substituted with alanine, and the mutated CaDRHB12 proteins were subsequently subjected to an in vitro kinase assay (Figure 5D). Compared with those of the intact CaDRHB12, the Ser15A and Ser142A substitution mutants showed reduced phosphorylation signals, with the decrease being more pronounced in the Ser15A mutant than in the Ser142A mutant. Additionally, the Ser15A/S142A double mutant displayed slightly lower phosphorylation levels than those in the Ser15A mutant. Conversely, Ser68A did not affect CaSnRK2.6‐mediated phosphorylation, as evidenced by the similar phosphorylation levels in the S142A single and Ser68A/S142A double mutants. These results suggest that Ser15 and Ser142 of CaDRHB12 are the main phosphorylation target sites of CaSnRK2.6.

### 3.6 CaDRHB12 Protein Degradation Is Modulated by CaSnRK2.6‐Mediated Phosphorylation in Response to ABA

Unlike CaDRHB12, CaSnRK2.6 plays a positive role in response to drought stress (Lim et al. 2021). Given the contrasting roles of CaDRHB12 and CaSnRK2.6, we investigated how CaSnRK2.6‐mediated phosphorylation influences the CaDRHB12 protein under drought‐stress conditions. In plants, protein phosphorylation is pivotal for modulating transcription factors, impacting their stability, subcellular localization, and activity in transcriptional activation (Li et al. 2016; Zhang et al. 2016; Maszkowska et al. 2019; Jiang et al. 2020). Thus, we first investigated the subcellular localization of the phosphorylation‐deficient mutants of CaDRHB12 (CaDRBH12^S15A/S142A^) to evaluate whether cellular localization was affected by phosphorylation (Figure S5). Microscopic analysis revealed that the mutated CaDRHB12 localised to the nucleus, similar to intact CaDRHB12 (Figure S5A). Moreover, this nuclear localization was not affected by ABA treatment and drought stress (Figure S5B), which promotes CaDRHB12 phosphorylation, indicating that phosphorylation does not impact CaDRHB12 localization.

**FIGURE 6:**
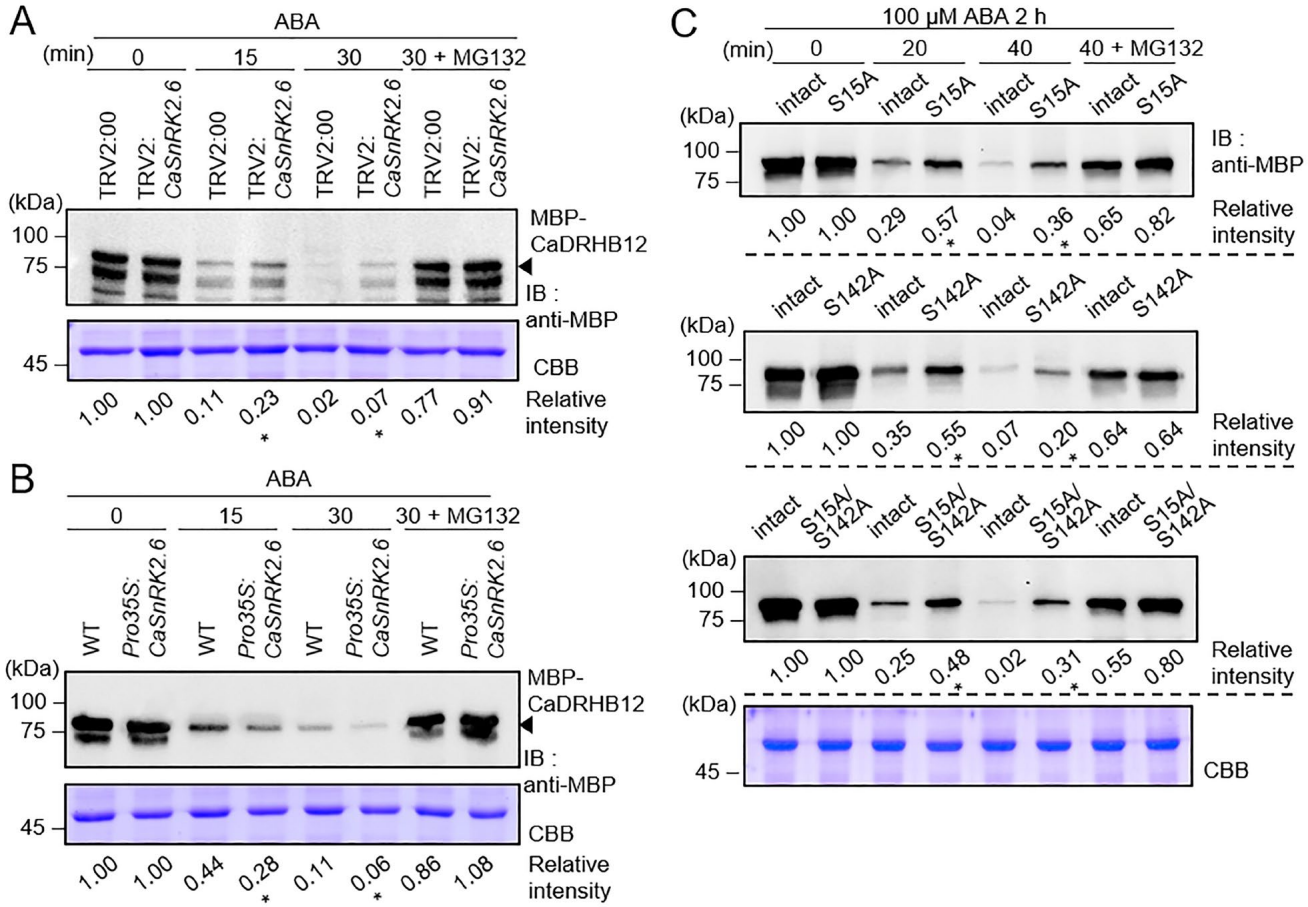
Modulation of CaDRHB12 protein degradation by CaSnRK2.6‐mediated phosphorylation in response to ABA. (A, B) Modulation of CaDRHB12 protein stability through *CaSnRK2.6* expression. For the cell‐free degradation assay, crude leaf extracts were prepared from the leaves of 3‐week‐old *CaSnRK2.6*‐silenced (TRV2:*CaSnRK2.6*) pepper plants (A) and *CaSnRK2.6*‐overexpressing *Arabidopsis* plants (B) treated with 100 μM ABA for 2 h and were then mixed with the MBP‐CaDRHB12 protein expressed in bacterial cells. The mixtures were incubated at the indicated time points, and MG132 was added as a 26S protease inhibitor. Coomassie brilliant blue (CBB) staining (below) indicated equal loading of the crude extracts. (C) Effect of serine residue substitution in CaDRHB12 protein on protein stability. Intact CaDRHB12 and its substitution variants CaDRHB12^S15A^, CaDRHB12^S142A^, and CaDRHB12^S15AS142A^ were incubated with crude leaf extracts from ABA‐treated pepper plants. Relative intensities were measured using ImageJ software (NIH), with pre‐incubation samples designated as the standard (1.00). All data represent the means ± SD of three independent experiments. Asterisks indicate significant differences between samples (Student's *t*‐test; *p < 0.05).

We further explored the effect of CaDRHB12 phosphorylation on its protein stability (Figure 6). Given that CaSnRK2.6 phosphorylates CaDRHB12, we speculated whether CaSnRK2.6 is involved in CaDRHB12 degradation in response to ABA. To verify this, a cell‐free degradation assay was performed with crude leaf extracts from TRV2:*CaSnRK2.6* pepper plants and *Pro35S:CaSnRK2.6 Arabidopsis* plants following ABA treatment (Figure 6A,B). Immunoblot analysis revealed that CaDRHB12 protein levels were higher following incubation with crude leaf extracts from TRV2:*CaSnRK2.6* plants than those from TRV2:00 plants (Figure 6A). In contrast, incubation with crude leaf extracts from *Pro35S:CaSnRK2.6* plants resulted in opposite patterns (Figure 6B). The degradation of CaDRHB12 protein was blocked by applying MG132, a 26S proteasome inhibitor, indicating that CaDRHB12 protein degradation through the 26S proteasome pathway may be associated with CaSnRK2.6 expression. We further investigated whether CaSnRK2.6‐mediated phosphorylation affects CaDRHB12 protein stability. The phosphorylation‐deficient CaDRHB12 mutants CaDRHB12 S15A, S142A and S15A/S142A were subjected to a cell‐free degradation assay (Figure 6C). After incubation with the crude extracts from ABA‐treated pepper leaves, all phosphorylation‐deficient mutants were less degraded than the intact CaDRHB12 protein. These results suggest that CaDRHB12 protein stability is regulated by CaSnRK2.6‐mediated phosphorylation in response to ABA.

### 3.7 Drought Stress Induces Phosphorylation‐Driven Regulation of CaDRHB12 by CaSnRK2.6

**FIGURE 7:**
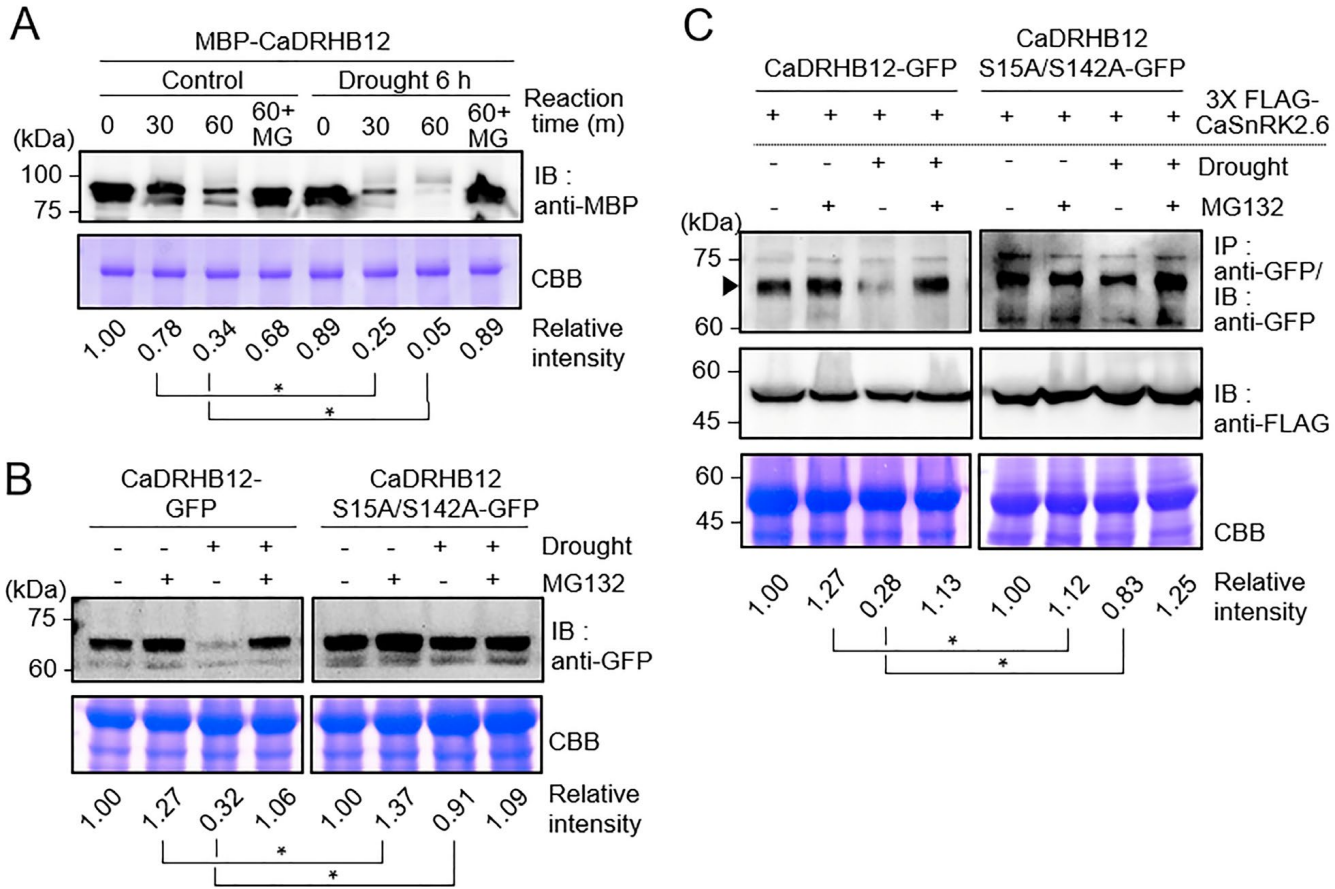
Modulation of CaDRHB12 protein degradation by CaSnRK2.6‐mediated phosphorylation in response to drought stress. (A) Rapid degradation of CaDRHB12 protein under drought stress conditions. For the cell‐free degradation assay, crude leaf extracts from pepper leaves dried for 6 h and control leaves were mixed with MBP‐CaDRHB12 protein expressed in bacterial cells. The mixtures were incubated at the indicated time points, and MG132 was added as a 26S protease inhibitor. (B and C) Effect of drought stress on the protein stability of intact CaDRHB12 and its phosphorylation‐deficient mutant CaDRHB12^S15AS142A^. *Nicotiana benthamiana* plants transiently expressing *CaDRHB12‐GFP* and *CaDRHB12* ^*S15AS142A*^ *‐GFP* were exposed to drought stress by removing their roots and drying for 30 min before sampling. MG132 (50 μM) was infiltrated into the inoculated leaves 12 h before drought stress treatment. Under the same conditions, *3×FLAG‐CaSnRK2.6* was additionally co‐expressed to examine the effects of *CaSnRK2.6* expression on CaDRHB12 protein degradation (C). Coomassie brilliant blue (CBB) staining indicated equal loading of the crude extracts. Relative intensities were measured using ImageJ software (NIH), with pre‐incubation samples designated as the standard (1.00). All data represent the means ± SD of three independent experiments. Asterisks indicate significant differences between samples (Student's *t*‐test; *p < 0.05).

As shown in Figure 5B, CaDRHB12 proteins were phosphorylated under drought stress and ABA treatment; hence, we examined how drought stress affects CaDRHB12 protein stability. A cell‐free degradation assay was performed by incubating MBP‐tagged CaDRBH12 proteins with crude leaf extracts from pepper plants subjected to drought stress (Figure 7A). We found that MBP‐tagged CaDRHB12 proteins degraded more rapidly when incubated with crude extracts from drought‐treated leaves than with those from control leaves. Specifically, after incubation for 1 h, 34% of CaDRHB12 proteins remained in the control sample, whereas almost all CaDRHB12 proteins were degraded in the drought stress sample. This degradation was blocked by MG132, indicating that drought stress facilitates CaDRHB12 protein degradation through the 26S proteasome pathway.

Additionally, we examined the effects of drought stress on CaDRHB12 protein degradation *in planta* (Figure 7B,C). To this end, we exposed tobacco plants transiently overexpressing *CaDRHB12‐GFP* to drought stress by removing soil from their roots and drying them for 30 min before harvesting the inoculated leaf samples. Immunoblot analysis showed a significant reduction in CaDRHB12 protein levels after drought stress treatment (Figure 7B, left). This reduction was not due to transcriptional differences in the *CaDRHB12* gene across samples (Figure S6). To further assess the effect of phosphorylation on CaDRHB12 protein stability under drought stress, we transiently expressed a phosphorylation‐deficient mutant, CaDRHB12^S15A/S142A^ (Figure 7B, right). The results showed that the CaDRHB12^S15A/S142A^ protein levels remained significantly higher than those of intact CaDRHB12: approximately 70% of intact CaDRHB12 underwent degradation under drought stress, whereas only 10% of the CaDRHB12^S15A/S142A^ mutant was degraded. Given that CaSnRK2.6 phosphorylates CaDRHB12 and that its kinase activity is activated by drought stress (Figure 5), we next co‐expressed 3×FLAG‐CaSnRK2.6 with either CaDRHB12‐GFP or CaDRHB12^S15A/S142A^ GFP in tobacco leaves and then repeated the drought stress treatment. Co‐expression did not alter basal stability, but drought still selectively triggered degradation of the intact CaDRHB12, not the mutant (Figure 7C). These findings suggest that CaSnRK2.6‐mediated phosphorylation is required for drought‐induced CaDRHB12 destabilisation.

### 3.8 CaDRHB12 Transcription Factor Activity Is Regulated by CaSnRK2.6‐Mediated Phosphorylation

**FIGURE 8:**
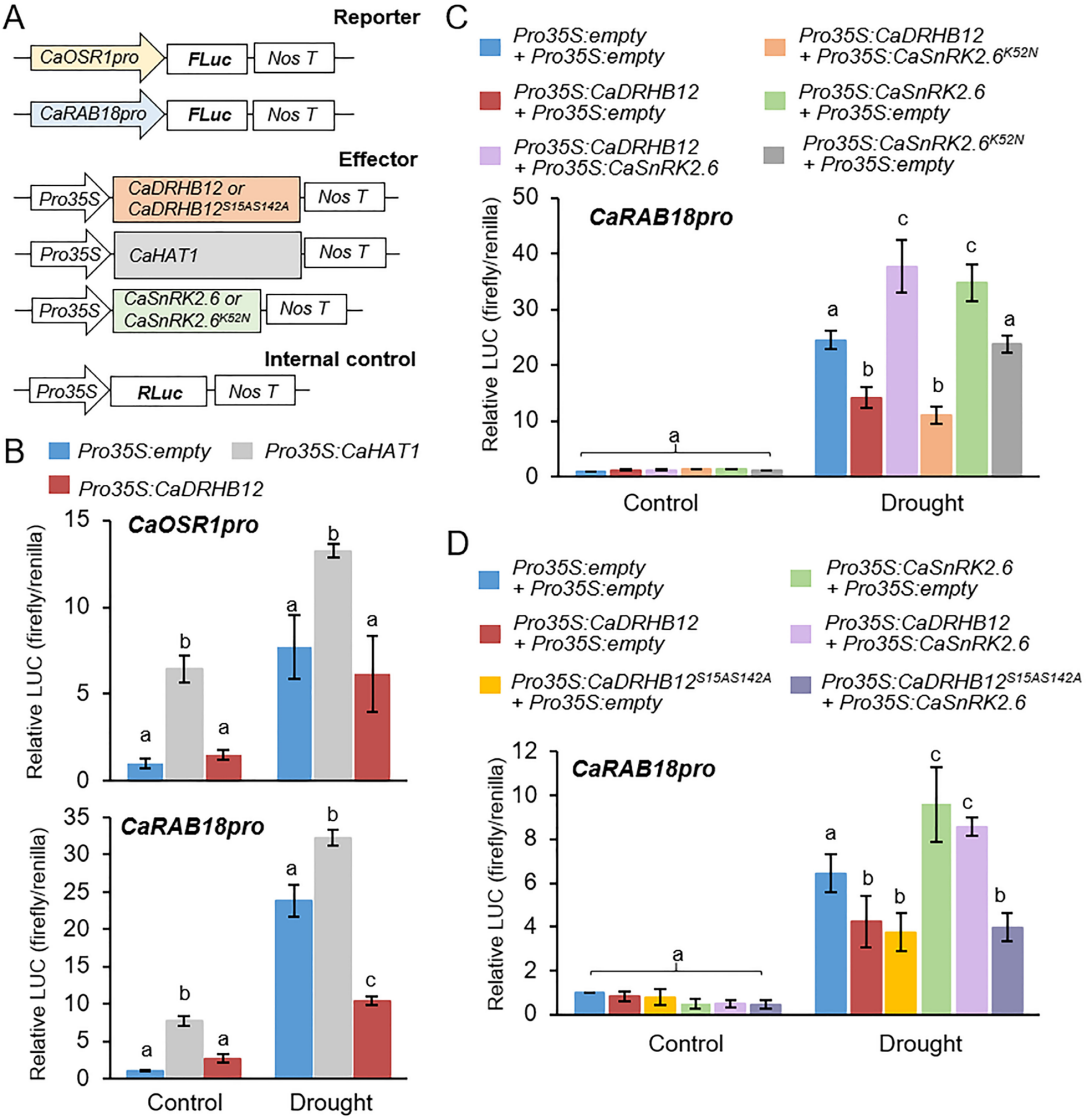
Transactivation of the pepper stress‐marker gene *CaRAB18* promoter‐driven luciferase (LUC) by CaDRHB12 in response to drought stress. (A) Schematic representation of the vector constructs used as reporters, effectors, and internal controls in the dual‐luciferase reporter assay. (B) Effect of CaDRHB12 on the activation of *CaOSR1‐* and *CaRAB18‐*promoter‐driven luciferase in response to drought stress. *Nicotiana benthamiana* leaves were co‐transformed with combinations of reporter and effector constructs, then subjected to drought stress by detachment and air‐drying for 30 min. Along with intact CaDRHB12, CaHAT1—a positive regulator of drought response—was included as a positive control to compare their roles in regulating *CaOSR1*‐ and *CaRAB18*‐promoter‐driven LUC expression. (C, D) Effect of CaDRHB12 phosphorylation by CaSnRK2.6 on the activation of *CaOSR1‐* and *CaRAB18‐*promoter‐driven luciferase in response to drought stress. In *Nicotiana benthamiana* leaves, CaDRHB12 was co‐transformed with either CaSnRK2.6 or CaSnRK2.6^K52N^ (C). Alternatively, intact CaDRHB12 or its phospho‐deficient mutant CaDRHB12^S15AS142A^ was co‐transformed with CaSnRK2.6 (D). The relative Firefly LUC/*Renilla* (REN) LUC fluorescence ratio was determined using the reporter system. The value for the empty vector in the control sample was set to 1. Data represent the mean ± SE of three independent experiments. Different letters indicate significant differences between treatments (ANOVA; *p* < 0.05).

Given the DNA‐binding activity in the C‐terminal region of CaDRHB12 in yeast cells (Figure 1B) and its effect on the expression of drought‐responsive genes, such as *CaOSR1* and *CaRAB18*, in plants (Figure 2G), we hypothesised that CaDRHB12 could act as a transcription factor that regulates these two genes in response to drought stress. To test this hypothesis, we conducted a dual‐luciferase reporter assay to determine whether CaDRHB12 binds to the upstream region of *CaOSR1* and *CaRAB18* genes (Figure 8). As shown in Figure 8A, various combinations of CaDRHB12, CaDRBH12^S15A/S142A^ mutants, and CaSnRK2.6 as effectors, along with the promoter of *CaOSR1* and *CaRAB18* genes, were transiently expressed in tobacco plants. These plants were subsequently subjected to drought stress, as described above. Consistent with previous data (Baek et al. 2023), CaHAT1 facilitated high luciferase expression driven by both *CaOSR1* and *CaRAB1* promoters before and after drought treatment compared with that of the empty vector control (Figure 8B). Unlike CaHAT1, CaDRHB12 could not activate or suppress this gene expression under normal conditions: the luciferase expression levels were similar to those of the control. Notably, after drought stress treatment, luciferase expression driven by the *CaRAB18* promoter and mediated by CaDRHB12 significantly decreased, whereas luciferase activity under the control of the *CaOSR1* promoter showed no significant difference relative to the vector control. These results suggest that CaDRHB12 may bind to *CaRAB18* promoters under drought stress conditions, thereby suppressing *CaRAB18* gene expression.

Next, we investigated whether CaDRHB12 phosphorylation contributes to suppressing *CaRAB18* expression. For the dual‐luciferase reporter assay, *CaSnRK2.6* and its kinase‐dead mutant, *CaSnRK2.6* ^*K52N*^, were co‐overexpressed with *CaDRHB12* in tobacco plants, and the *CaRAB18* promoter was used as a reporter (Figure 8C). Under normal conditions, luciferase expression levels mediated by *CaDRHB12* and/or *CaSnRK2.6* did not significantly differ. However, after drought stress treatment, the *CaDRHB12*‐mediated suppression of luciferase expression was blocked by *CaSnRK2.6* expression, resulting in expression levels similar to those mediated by the expression of *CaSnRK2.6* alone without *CaDRHB12*. However, the reduction of luciferase expression was not induced by the *CaSnRK2.6* ^*K52N*^ mutant. To further clarify the influence of CaDRHB12 phosphorylation status on its transactivation, we used a phosphorylation‐deficient mutant, *CaDRHB12* ^*S15A/S142A*^, and expressed it with *CaSnRK2.6* in tobacco plants (Figure 8D). Similar to that of intact *CaDRHB12*, *CaDRHB12* ^*S15A/S142A*^ expression led to a significant reduction in *CaRAB18* promoter‐driven luciferase expression levels under drought stress conditions. This pattern was not affected by co‐expression with *CaSnRK2.6*. These data suggest that the CaSnRK2.6‐mediated phosphorylation of CaDRHB12 contributes to enhancing *CaRAB18* expression under drought stress conditions.

### 3.9 CaDRHB12 Acts Downstream of CaSnRK2.6 in Response to Drought Stress

**FIGURE 9:**
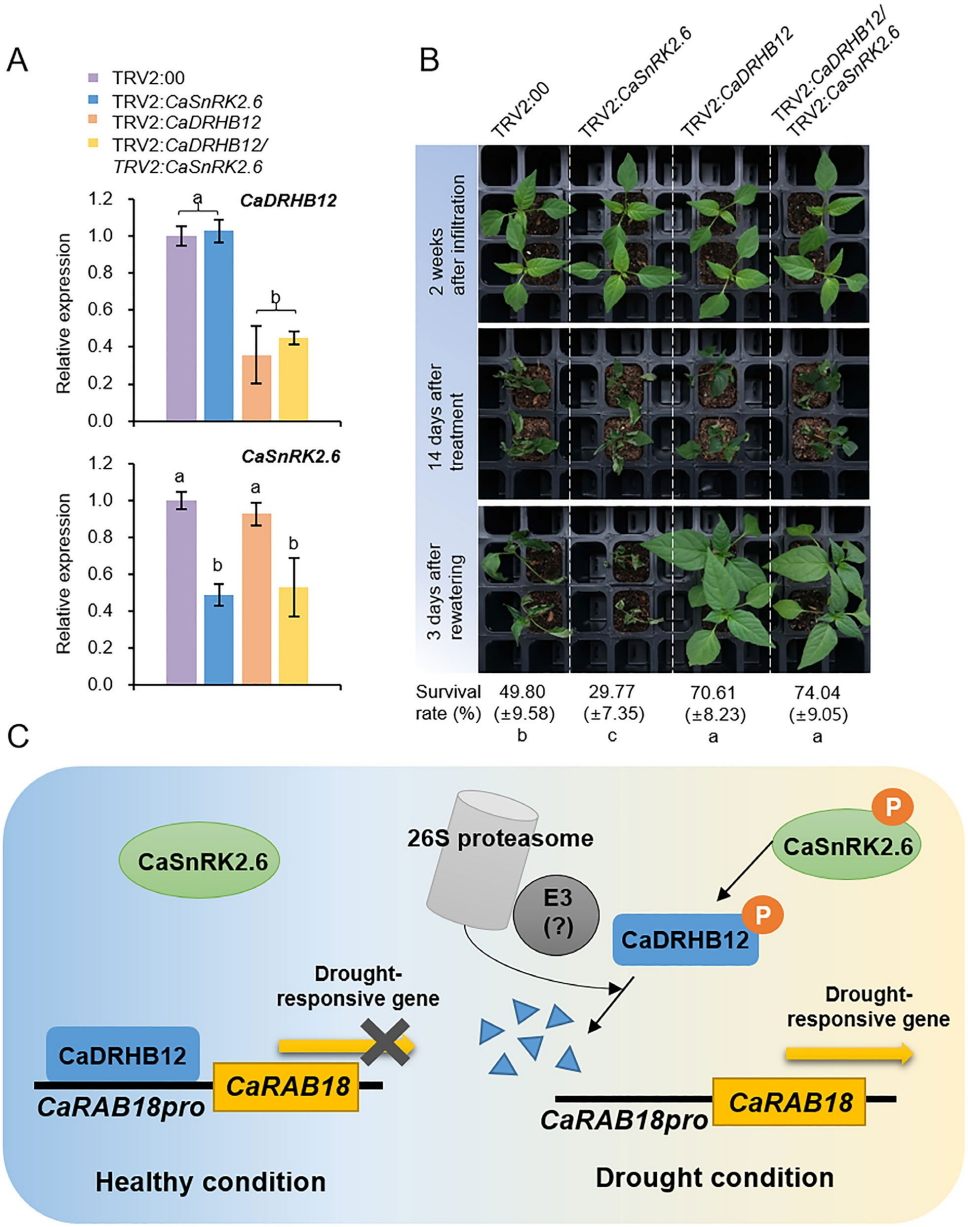
Functional interplay relationship between *CaDRHB12* and *CaSnRK2.6* genes in response to drought stress. (A) *CaDRHB12* and *CaSnRK2.6* expression levels in gene‐silenced pepper plants. RT‐qPCR analysis was conducted to evaluate *CaDRHB12* and *CaSnRK2.6* transcript levels in TRV2:00, TRV2:*CaDRHB12*, TRV2:*CaSnRK2.6*, and TRV2:*CaDRHB12/CaSnRK2.6* pepper plants. (B) Enhanced drought tolerance of TRV2:*CaDRHB12/CaSnRK2.6* pepper plants. TRV2:00, TRV2:*CaDRHB12*, TRV2:*CaSnRK2.6*, and TRV2:*CaDRHB12/CaSnRK2.6* pepper plants grown for 2 weeks after agroinfiltration were exposed to drought stress by growing them without water for 14 days. Representative images were obtained before (top) and after (middle) drought treatment. At 3 days after rewatering (bottom), the survival rates were evaluated by counting the number of surviving plants. Different letters indicate significant differences between three independent experiments (ANOVA; *p* < 0.05). (C) A proposed model highlighting the function of the CaSnRK2.6‐CaDRHB12‐CaRAB18 regulatory module in modulating the drought stress response in pepper plants. Under normal conditions, CaDRHB12 acts as a repressor of *CaRAB18* gene expression, potentially by binding to its promoter. Upon exposure to drought stress, CaSnRK2.6 becomes activated and phosphorylates its downstream target, CaDRHB12. The phosphorylated CaDRHB12 is subsequently destabilised and degraded via the 26S proteasome pathway, mediated by E3 ligases that have yet to be identified. The degradation of CaDRHB12, a transcriptional repressor, alleviates its inhibitory effect on *CaRAB18* expression, facilitating a robust drought stress response in pepper plants.

To explore the functional relationship between CaDRHB12 and CaSnRK2.6 in response to drought stress, we generated double gene‐knock‐down pepper (TRV2:*CaDRHB12*/TRV2:*CaSnRK2.6*) using the VIGS method (Figure 9). RT‐qPCR analysis demonstrated a significant reduction in the expression levels of both *CaDRHB12* and *CaSnRK2.6* in TRV2:*CaDRHB12*/TRV2:*CaSnRK2.6* plants compared with those in the control TRV2:00 (Figure 9A). Next, we evaluated the drought tolerance of these plants as well as that of TRV2:00, TRV2:*CaSnRK2.6*, and TRV2:*CaDRHB12* plants. After 2 weeks of agroinfiltration, all four plant lines were subjected to drought stress by withholding water for 2 weeks, followed by rewatering for 3 days (Figure 9B). TRV2:*CaDRHB12* pepper plants showed a much higher survival rate (approximately 70.61%) under drought stress compared with that of TRV2:00 plants (49.8%), whereas only 29.7% of TRV2:*CaSnRK2.6* plants survived. Notably, silencing both *CaDRHB12* and *CaSnRK2.6* led to enhanced drought tolerance, similar to that observed in TRV2:*CaDRHB12* pepper plants. These results suggest that CaDRHB12 functions downstream of CaSnRK2.6 under drought stress conditions. In this process, the activated CaSnRK2.6 kinase phosphorylates CaDRHB12, prompting its degradation and, consequently, regulating the drought stress response.

## 4 Discussion

The HD‐ZIP family, a plant‐specific group of transcription factors, plays a critical role in regulating plant growth, development, and response to abiotic stresses, particularly drought (Lim, Hong, et al. 2018; Li et al. 2022). Increasing evidence suggests that their activity is tightly modulated by post‐translational modifications (PTMs), including phosphorylation, ubiquitination, and sumoylation (Harris et al. 2011; Lechner et al. 2011; Zhang et al. 2014; Tan et al. 2018; Joo et al. 2022). In the present study, we identified and characterised CaDRHB12, an HD‐ZIP II transcription factor in pepper, as a negative regulator of drought stress. Functional analyses revealed that silencing the *CaDRHB12* gene enhanced drought tolerance in pepper, while overexpression in Arabidopsis increased drought sensitivity (Figures 2 and 3), suggesting a conserved inhibitory role.

Mechanistically, we demonstrated that CaDRHB12 represses the expression of *CaRAB18*, a known drought‐responsive gene (Figure 8). This repression is alleviated upon interaction with CaSnRK2.6, a drought‐responsive Ser/Thr protein kinase. CaSnRK2.6‐mediated phosphorylation promotes CaDRHB12 degradation via the 26S proteasome pathway, relieving repression of downstream drought‐responsive genes (Figure 9C). These findings suggest that CaDRHB12 acts as a molecular brake, which is removed under drought conditions through targeted phosphorylation and degradation, thereby enabling the activation of stress response genes.

The functional involvement of HD‐ZIP class II genes in abiotic stress responses has been reported across various plant species. In *Arabidopsis*, the HD‐ZIP class II gene *HAT1* negatively regulates drought tolerance (Tan et al. 2018), whereas the *Eucalyptus* HD‐ZIP II gene *EcHB1* plays a positive role in drought tolerance (Sasaki et al. 2019). Similarly, the heterologous expression of the pepper HD‐ZIP II gene *CaHB1* in tomato enhanced tolerance against salt stress (Oh et al. 2013). Our previous study demonstrated that *CaHAT1*, a member of the HD‐ZIP II transcription factor family, plays a positive role in drought tolerance (Baek et al. 2023), while CaDRHB12 negatively regulates drought responses. Sequence alignment analysis showed that the CaHAT1 protein shares 66% identity and 72.9% similarity with CaDRHB12, both of which exhibit the characteristic domain architecture of the HD‐ZIP II family. This includes the HD‐ZIP N‐terminal region, homeobox (HOX) domain, HALZ domain, and characteristic EAR and CPSCE motifs (Figure S1). Despite their high sequence homology and shared HD‐ZIP II domain architecture, CaDRHB12 and CaHAT1 exhibit functionally divergent roles. Notably, both CaDRHB12 and CaHAT1 proteins interacted with and were phosphorylated by CaSnRK2.6 kinase. However, their phosphorylation sites appear to differ significantly. In CaHAT1, Ser92 was identified as a critical residue for CaSnRK2.6‐mediated phosphorylation. In contrast, Ser15 was essential for CaDRHB12 phosphorylation. Interestingly, Ser108 of CaDRHB12, which corresponds to Ser92 in CaHAT1, did not appear to contribute to CaDRHB12 phosphorylation by CaSnRK2.6; mutation of Ser108 in CaDRHB12 did not affect phosphorylation levels (Figure S8). These findings suggest that CaSnRK2.6 differentially regulates CaHAT1 and CaDRHB12 by targeting distinct serine residues for phosphorylation, thereby contributing to their opposing roles in drought stress responses.

The CaSnRK2.6‐mediated phosphorylation of CaDRHB12 and CaHAT1 proteins was activated by treatment with ABA and drought stress. Notably, these treatments affected the stability of CaDRHB12 and CaHAT1 proteins differentially. A cell‐free degradation assay demonstrated that ABA and drought stress promoted the degradation of CaDRHB12, whereas they delayed the degradation of CaHAT1 (Figure S9). CaDRHB12 protein degradation was shown to be associated with its phosphorylation state: Ser15 and Ser142 mutation significantly inhibited CaDRHB12 protein degradation in response to ABA and drought stress. As a member of the *Arabidopsis* HD‐ZIP II transcription factor family, HAT1shares 55% identity and 63.3% similarity in the amino acid sequence with CaDRHB12 and also functions as a negative regulator of ABA signalling and drought response. Notably, HAT1 undergoes phosphorylation mediated by SnRK2.3, and this phosphorylation, which is activated by ABA treatment, promotes its destabilisation (Tan et al. 2018). Based on these findings, the phosphorylation‐dependent regulation of HD‐ZIP II transcription factors by SnRK2 kinases may be a conserved mechanism in plants, influencing the stability and functional roles of HD‐ZIP II transcription factors in response to ABA and drought stress. Moreover, our results suggest that CaSnRK2.6 mediates a dual regulatory mechanism: promoting drought tolerance by stabilising transcription factors such as CaHAT1 while simultaneously degrading negative regulators such as CaDRHB12. This 'two‐trac' regulatory mechanism enables a balanced and efficient drought stress response.

Expression analysis further supports the divergent roles of CaDRHB12 and CaHAT1 proteins. The modulation of drought tolerance by CaDRHB12 and CaHAT1 was accompanied by the differential expression of drought‐responsive genes, including *CaOSR1* and *CaRAB18*. Specifically, the expression of *CaOSR1* and *CaRAB18* was increased in *CaDRHB12*‐silenced pepper plants under drought stress (Figure 2G), whereas silencing of *CaHAT1* led to a significant decrease in their expression levels (Baek et al. 2023). This divergence in regulatory patterns may stem from two mechanisms. First, the differential modulation of CaHAT1 and CaDRHB12 protein stability under drought conditions likely underpins their distinct regulatory effects (Figures 7 and S9), although the precise mechanisms remain to be elucidated. Second, our dual‐luciferase assay results highlighted functional differences between CaDRHB12 and CaHAT1. Co‐expression of CaHAT1 significantly increased luciferase expression driven by the *CaOSR1* and *CaRAB18* promoters, an effect further amplified by the presence of active CaSnRK2.6 kinase under drought conditions (Baek et al. 2023). In contrast, CaDRHB12 inhibited luciferase expression driven by the *CaRAB18* promoter, but this repression was significantly reversed by co‐expression of active *CaSnRK2.6*, restoring luciferase expression to levels comparable to those observed with *CaSnRK2.6* alone (Figure 8). Interestingly, despite the elevated expression of *CaOSR1* in *CaDRHB12*‐silenced pepper plants under drought stress, no corresponding change in *CaOSR1* promoter‐driven luciferase activity was observed in the presence of CaDRHB12. This suggests that *CaOSR1* may be regulated through indirect or independent pathways under drought conditions, though the exact mechanism is yet to be determined. EMSA analysis provided one possible explanation for this difference. Sequence alignment revealed that the ATHB6COREAT motif (CAATTATTA) is present specifically in the *CaRAB18* promoter but not in the *CaOSR1* promoter. Notably, CaDRHB12 directly binds to this motif in the *CaRAB18* promoter, as shown by EMSA (Figure S7), supporting the results of our dual‐luciferase assays (Figure 8). Unlike *Arabidopsis* HAT1, which directly represses ABA biosynthesis genes such as *ABA3* and *NCED3* (Tan et al. 2018), neither silencing nor overexpression of CaDRHB12 or CaHAT1 affected *NCED3* expression in pepper or *Arabidopsis* plants. These findings indicate that CaDRHB12 and CaHAT1 play distinct roles in drought response, differing from those of *Arabidopsis* HAT1. Specifically, they regulate the expression of drought‐responsive genes rather than influencing ABA biosynthesis.

In conclusion, this study provides the first mechanistic evidence of differential phosphorylation targeting by CaSnRK2.6 leading to opposing regulatory outcomes for closely related HD‐ZIP II transcription factors in pepper. We demonstrate that CaDRHB12 functions as a negative regulator of drought stress in pepper plants. Upon exposure to drought stress, CaDRHB12 phosphorylation by CaSnRK2.6 promotes its degradation via the 26S proteasome pathway, thereby promoting the expression of downstream drought‐responsive genes and enhancing drought tolerance. Together with our findings on CaHAT1, we propose a CaSnRK2.6‐centered regulatory module that dynamically balances transcription factor activity—stabilising positive regulators while degrading repressors—to optimise drought stress responses. To further elucidate the regulatory mechanisms underlying the CaSnRK2.6‐CaDRHB12/CaHAT1 module, future research should investigate the role of ubiquitination, particularly the involvement of ubiquitin E3 ligases, in the proteasomal degradation of CaDRHB12 and CaHAT1. Additionally, exploring other PTMs, such as sumoylation, will provide deeper insights into the complex regulatory networks governing HD‐ZIP II transcription factor activity during abiotic stress responses. These findings will contribute to a more comprehensive understanding of how HD‐ZIP II transcription factors are modulated through PTMs in response to abiotic stresses.

## Author Contributions

W.B., Y.B., C.W.L. and S.C.L.: conceptualization. W.B., Y.B. and C.W.L.: methodology. W.B. and Y.B.: data analysis. W.B., C.W.L. and S.C.L.: writing.

## Conflicts of Interest

The authors declare no conflicts of interest.

## Supporting information


**Figure S1:** Amino acid sequence alignment between CaHAT1 and CaDRHB12 using the EMBL‐EBI sequence analysis web tool (Clustal Omega, https://www.ebi.ac.uk/jdispatcher/msa/clustalo). Coloured underlines represent the functional domains and motifs of HD‐ZIP genes, and conserved motifs are indicated with different coloured underlines. CaDRHB12 shares 66% identity and 72.9% similarity with the CaHAT1 amino acid sequence.
**Figure S2:** Decreased ABA sensitivity of *CaDRHB12*‐silenced pepper plants. (A) Stomatal apertures of TRV2:*00* and TRV2:*CaDRHB12* pepper plants in the presence of ABA. Leaf peels harvested from 4‐week‐old plants of each line (*n* = 5) were incubated for 2 h in stomatal opening solution (SOS) containing 0, 10 or 20 μM ABA. Representative images were taken (left), and the stomatal apertures were measured using image J program (right). (B) Comparison in the leaf surface temperature between control and TRV2:*CaDRHB12* pepper plants in the presence of ABA or not. Four‐week‐old plants of each line (*n* = 15) were sprayed 100 μM ABA for 2 h. All data represents the means ± SD of three independent experiments.
**Figure S3:** Enhanced ABA sensitivity of *CaDRHB12*‐overexpressing *Arabidopsis* plants. (A) *CaDRHB12* expression levels. RT‐qPCR analysis was conducted to evaluate *CaDRHB12* transcript levels in wild‐type (WT) and *Pro35S:CaDRHB12 Arabidopsis* plants. Since the expression level of CaDRHB12 in WT was not detected (ND), those in *Pro35S:CaDRHB12* #13 samples were set to 1.0. Values are means ± SD of three independent experiments and different letters indicate significant differences (ANOVA: *p* < 0.05). (B) Seedling growth of *Pro35S:CaDRHB12* and WT plants in response to ABA. Seeds were germinated and grown vertically on 0.5× MS agar plates containing various ABA concentrations. After 7 days, representative images were obtained (upper panel), and the primary root length was measured (bottom panel). Data represent the mean ± SE of three independent experiments. Error bars represent the SE. Different letters indicate significant differences (ANOVA: *p* < 0.05). (C) ABA‐induced stomatal closure in WT and *Pro35S:CaDRHB12* plants. Stomatal apertures were measured 3 h after treatment with 0, 10 or 20 μM ABA. Representative images of the stomata were obtained for the leaves of each line (upper), and the apertures of 100 randomly selected stomata were measured (lower). (D) Leaf temperatures of WT and *Pro35S:CaDRHB12* plants after exposure to abscisic acid (ABA). Representative thermographic images were taken at 3 h after treatment with 100 μM ABA (upper) and the temperature were measured by the thermal image camera and calculated from the three largest leaves of plants from each line (*n* = 12) (lower).
**Figure S4:** Negative control images of the BiFC analysis presented in Figure 4C. To induce transient gene expression, *Agrobacterium* cells harbouring a combination of an empty vector and either CYCE‐CaSnRK2.6 or VYNE‐CaDRHB12 were infiltrated into *Nicotiana benthamiana* leaves. DAPI (blue signal) was used as a nuclear indicator. White bar = 20 μm.
**Figure S5:** Subcellular localization analysis. (A) Comparison of the subcellular localization of CaDRHB12 and its phosphorylation‐deficient mutant. *Pro35S:CaDRHB12‐GFP* and Pro35S:CaDRHB12^S15AS142A^‐GFP were transiently expressed in *Nicotiana benthamiana* leaves through agroinfiltration. (B) Analysis of the subcellular localization of CaDRHB12 in response to ABA and drought stress. Tobacco leaves transiently expressing CaDRHB12‐GFP were treated with 100 μM ABA for 6 h and dried for 60 min. GFP signals were observed 2 days after agroinfiltration using confocal microscopy. The blue fluorescence signal corresponds to the nuclear marker 4′, 6‐diamidino‐2‐phenylindole (DAPI). AtCENH3, fused with red fluorescent protein, served as a centromere marker. White bar = 20 μm (A) and 10 μm (B).
**Figure S6:** *CaDRHB12* expression levels in tobacco leaves expressing *CaDRHB12‐GFP* presented in Figure 7B (left panel). *NbPP2A* was used as an internal control in the RT‐qPCR analysis.
**Figure S7:** EMSA analysis of CaDRHB12 binding to the *CaRAB18* promoter region. (A) Schematic diagram of the *CaRAB18* promoter. The ATHB6COREAT motif is located around the 1555–1564 bp region. The lower panel shows the use of a biotin‐labelled probe and an unlabeled (cold) competitor probe. (B) EMSA showing binding of CaDRHB12 to AtHB6 interaction motif in the *CaRAB18* promoter in vitro. MBP‐CaDRHB12 protein was incubated with the biotin‐labelled probe. Binding competition was confirmed using an unlabeled cold probe. Protein input was verified by immunoblotting with an anti‐MBP antibody.
**Figure S8:** In vitro phosphorylation of CaDRHB12^S108A^ by CaSnRK2.6. For the in vitro kinase assay, CaDRHB12 and CaDRHB12^S108A^ were incubated with CaSnRK2.6. Coomassie brilliant blue (CBB) staining indicated protein input in the kinase assay.
**Figure S9:** Suppression of CaHAT1 protein degradation under ABA exposure and drought stress conditions. For the cell‐free degradation assay, crude leaf extracts from pepper leaves treated with 100 μM ABA for 2 h (upper panel) and dried for 6 h (bottom) were mixed with GST‐CaHAT1 protein expressed in bacterial cells. The mixtures were incubated at the indicated time points, and MG132 was added as a 26S protease inhibitor. Coomassie brilliant blue (CBB) staining indicated equal loading of the crude extracts. Relative intensities were measured using ImageJ software (NIH), with pre‐incubation samples designated as the standard (1.00). All data represent the means ± SD of three independent experiments. Asterisks indicate significant differences between samples (Student's *t*‐test; *p < 0.05).
**Table S1:** The sequences of primers used in this study.

## Data Availability

Data sharing is not applicable to this article as no new data were created or analyzed in this study.
