# Supplementary material for: Phosphorylation‐Induced Degradation of Pepper Dehydration‐Related Homeobox Gene 12 (CaDRHB12), a Negative Regulator of Drought Response in Pepper, by CaSnRK2.6 Enhances Drought Tolerance
Source: Plant Biotechnol J. 2025 Aug 6;23(11):5178–96. doi: 10.1111/pbi.70298 (PMC12576426; doi:10.1111/pbi.70298)
Supplement: Supplementary file 1 — Figure S1: Amino acid sequence alignment between CaHAT1 and CaDRHB12 using the EMBL‐EBI sequence analysis web tool (Clustal Omega, https://www.ebi.ac.uk/jdispatcher/msa/clustalo). Coloured underlines represent the functional domains and motifs of HD‐ZIP genes, and conserved motifs are indicated with different coloured underlines. CaDRHB12 shares 66% identity and 72.9% similarity with the CaHAT1 amino acid sequence. Figure S2: Decreased ABA sensitivity of CaDRHB12‐silenced pepper plants. (A) Stomatal apertures of TRV2:00 and TRV2:CaDRHB12 pepper plants in the presence of ABA. Leaf peels harvested from 4‐week‐old plants of each line (n = 5) were incubated for 2 h in stomatal opening solution (SOS) containing 0, 10 or 20 μM ABA. Representative images were taken (left), and the stomatal apertures were measured using image J program (right). (B) Comparison in the leaf surface temperature between control and TRV2:CaDRHB12 pepper plants in the presence of ABA or not. Four‐week‐old plants of each line (n = 15) were sprayed 100 μM ABA for 2 h. All data represents the means ± SD of three independent experiments. Figure S3: Enhanced ABA sensitivity of CaDRHB12‐overexpressing Arabidopsis plants. (A) CaDRHB12 expression levels. RT‐qPCR analysis was conducted to evaluate CaDRHB12 transcript levels in wild‐type (WT) and Pro35S:CaDRHB12 Arabidopsis plants. Since the expression level of CaDRHB12 in WT was not detected (ND), those in Pro35S:CaDRHB12 #13 samples were set to 1.0. Values are means ± SD of three independent experiments and different letters indicate significant differences (ANOVA: p < 0.05). (B) Seedling growth of Pro35S:CaDRHB12 and WT plants in response to ABA. Seeds were germinated and grown vertically on 0.5× MS agar plates containing various ABA concentrations. After 7 days, representative images were obtained (upper panel), and the primary root length was measured (bottom panel). Data represent the mean ± SE of three independent experiments. Error bars represent th [file PBI-23-5178-s001.pdf]

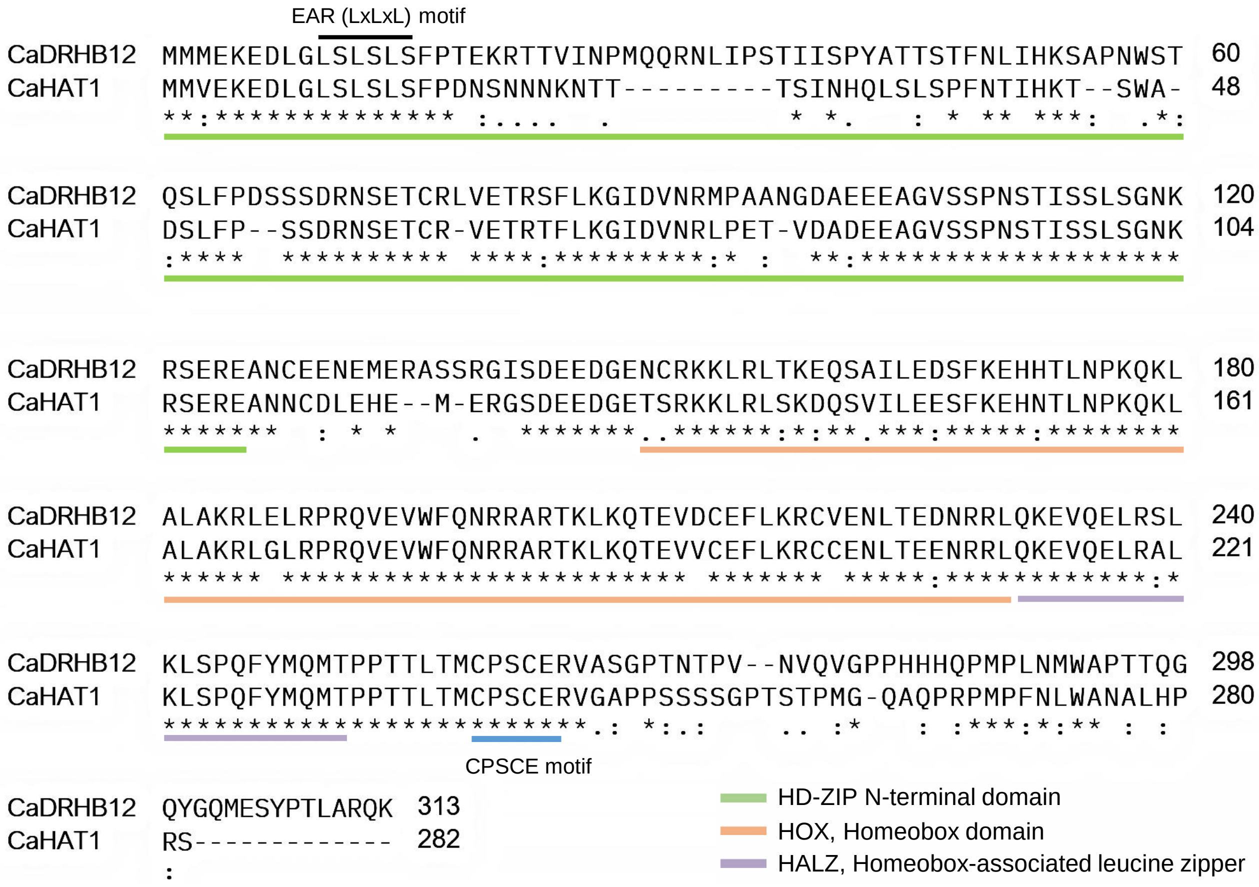

Supplementary Figure S1. Amino acid sequence alignment between CaHAT1 and CaDRHB12 using the EMBL-EBI sequence analysis web tool (Clustal Omega, https://www.ebi.ac.uk/jdispatcher/msa/clustalo). Colored underlines indicate the functional domains and motifs of HD-ZIP genes, with each motif represented by a distinct color. Amino acid sequence comparison reveals that CaDRHB12 shares 66% identity and 72.9% similarity with CaHAT1.

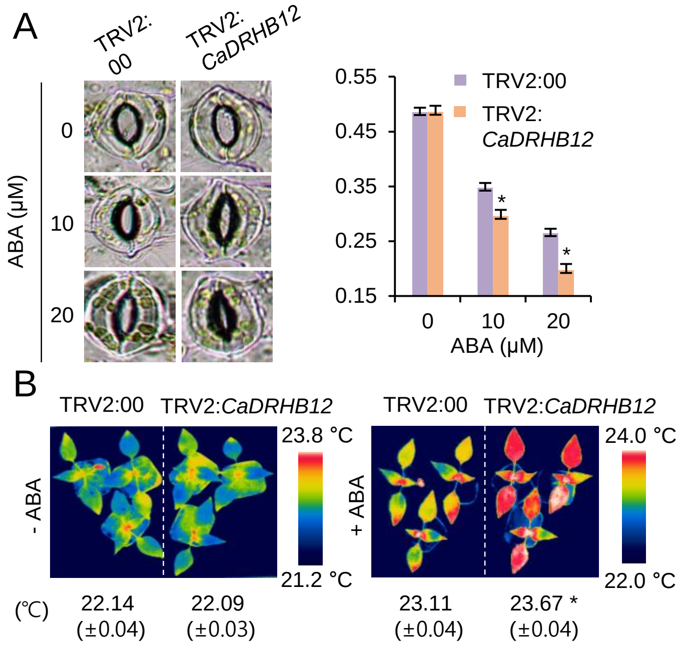

Supplementary Figure S2. Decreased ABA sensitivity of *CaDRHB12*-silenced pepper plants. (A) Stomatal apertures of TRV2:00 and TRV2:*CaDRHB12* pepper plants in presence of ABA. Leaf peels harvested from 4-week-old plants of each line (n=5) were incubated for 2 hours in stomatal opening solution (SOS) containing 0, 10 or 20 μM ABA. Representative images were taken (left) and the stomatal apertures were measured using image J program (right). (B) Comparison in leaf surface temperature between control and TRV2:*CaDRHB12* pepper plants in presence of ABA or not. Four-week-old plants of each line (n=15) were sprayed 100 μM ABA for 2 h. All data represents the means ± SD of three independent experiments.

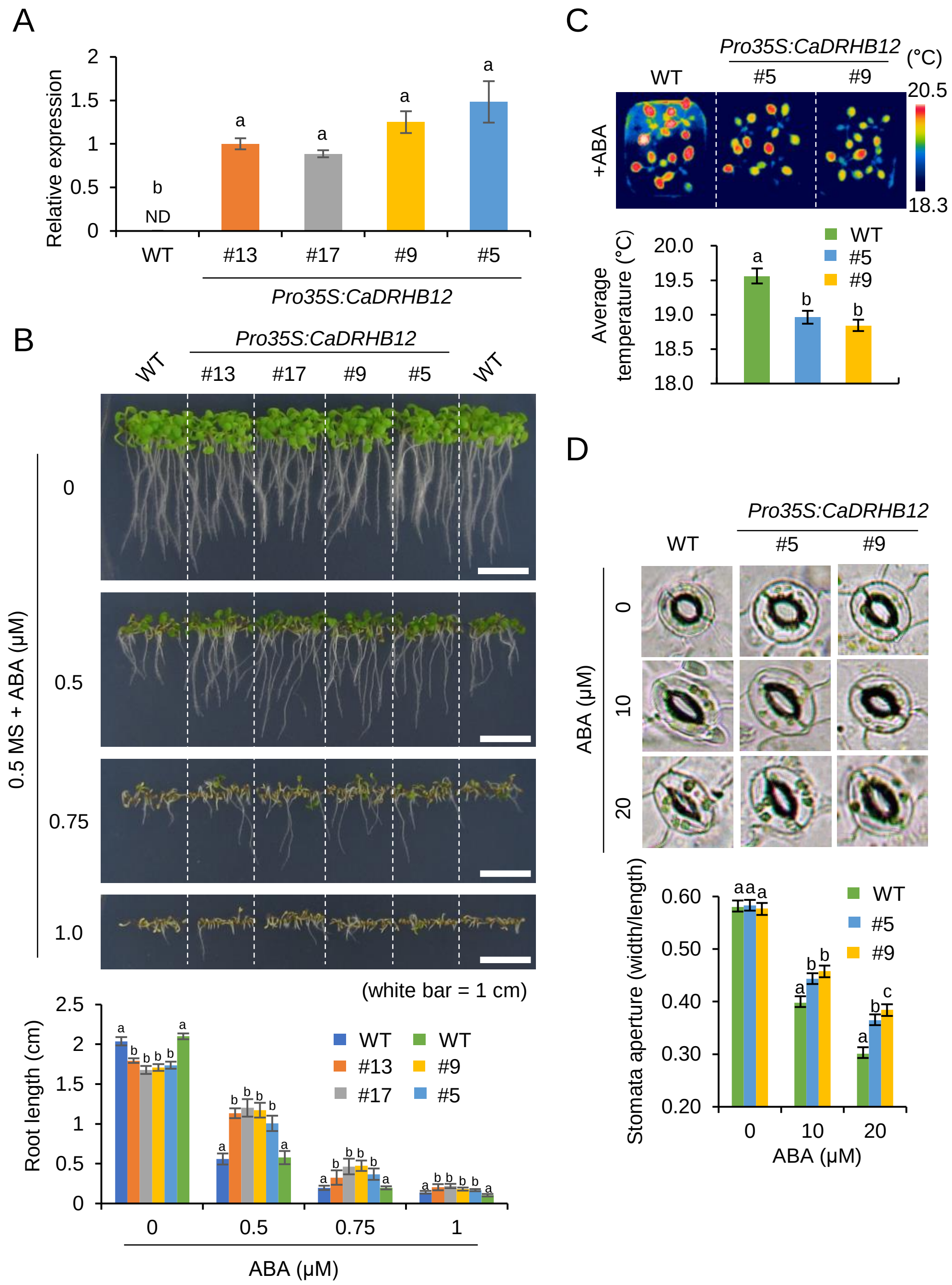

Supplementary Figure S3. Enhanced ABA sensitivity of *CaDRHB12*-overexpressing *Arabidopsis* plants. (A) *CaDRHB12* expression levels. RT-qPCR analysis was conducted to evaluate *CaDRHB12* transcript levels in wild-type (WT) and *Pro35S:CaDRHB12 Arabidopsis* plants. Since the expression level of CaDRHB12 in WT was not detected (ND), those in *Pro35S:CaDRHB12* #13 samples were set to 1.0. Values are means ± standard deviation of three independent experiments and different letters indicate significant differences (ANOVA: $P < 0.05$). (B) Seedling growth of *Pro35S:CaDRHB12* and WT plants in response to ABA. Seeds were germinated and grown vertically on 0.5× MS agar plates containing various ABA concentrations. After 7 days, representative images were obtained (upper panel), and the primary root length was measured (bottom panel). Data represent the mean ± SE of three independent experiments. Error bars represent the SE. Different letters indicate significant differences (ANOVA: $P < 0.05$). (C) ABA-induced stomatal closure in WT and *Pro35S:CaDRHB12* plants. Stomatal apertures were measured 3 h after treatment with 0, 10, or 20 μM ABA. Representative images of the stomata were obtained for the leaves of each line (upper) and the apertures of 100 randomly selected stomata were measured (lower). (D) Leaf temperatures of WT and *Pro35S:CaDRHB12* plants after exposure to abscisic acid (ABA). Representative thermographic images were taken at 3 h after treatment with 100 μM ABA (upper) and the temperature were measured by the thermal image camera and calculated from the three largest leaves of plants from each line (n = 12) (lower).

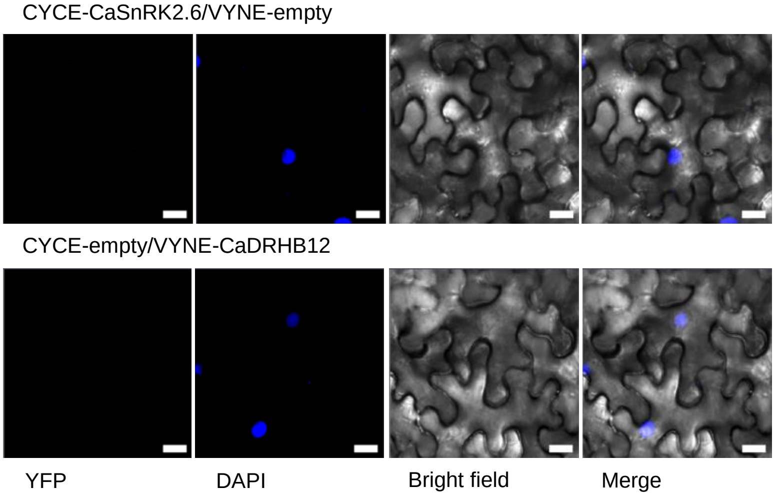

Supplementary Figure S4. Negative control images of the BiFC analysis presented in Figure 4C. To induce transient gene expression, *Agrobacterium* cells harboring a combination of an empty vector and either CYCE-CaSnRK2.6 or VYNE-CaDRHB12 were infiltrated into *Nicotiana benthamiana* leaves. DAPI (blue signal) was used as a nuclear indicator. White bar = 20 μm.

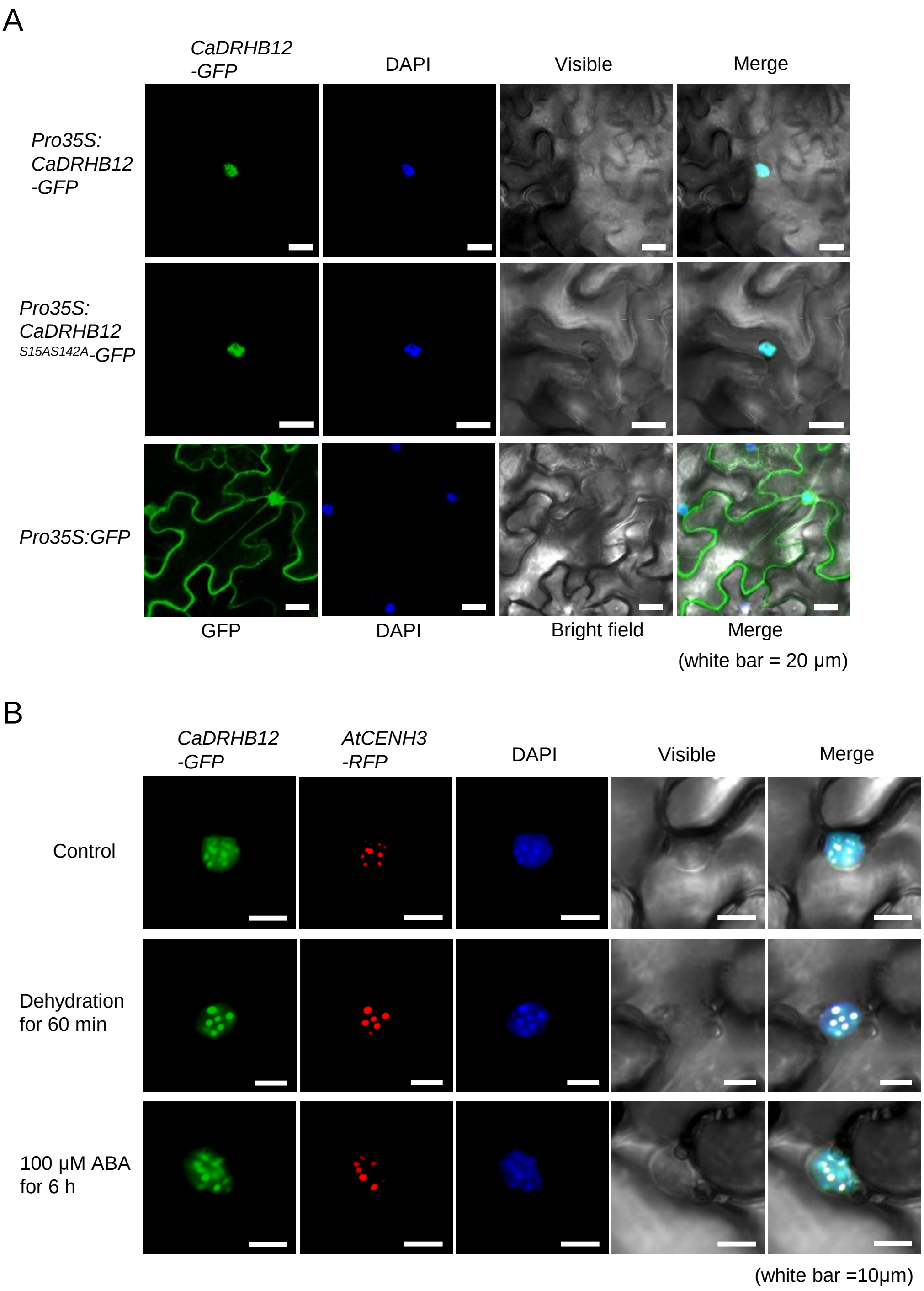

Supplementary Figure S5. Subcellular localization analysis. (A) Comparison of the subcellular localization of CaDRHB12 and its phosphorylation-deficient mutant. *Pro35S:CaDRHB12-GFP* and Pro35S:CaDRHB12^{S15AS142A}-GFP were transiently expressed in *Nicotiana benthamiana* leaves through agroinfiltration. (B) Analysis of the subcellular localization of CaDRHB12 in response to ABA and drought stress. Tobacco leaves transiently expressing CaDRHB12-GFP were treated with 100 µM ABA for 6 h and dried for 60 min. GFP signals were observed 2 days after agroinfiltration using confocal microscopy. The blue fluorescence signal corresponds to the nuclear marker 4′, 6-diamidino-2-phenylindole (DAPI). AtCENH3, fused with red fluorescent protein, served as a centromere marker. White bar = 20 µm (A) and 10 µm (B).

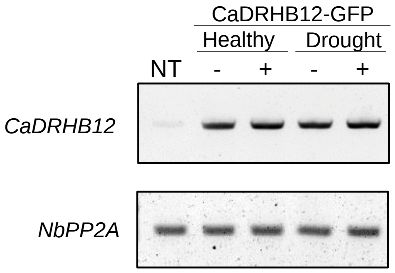

Supplementary Figure S6. *CaDRHB12* expression levels in tobacco leaves expressing *CaDRHB12-GFP* presented in Figure 7B (left panel). *NbPP2A* was used as an internal control in the RT-qPCR analysis.

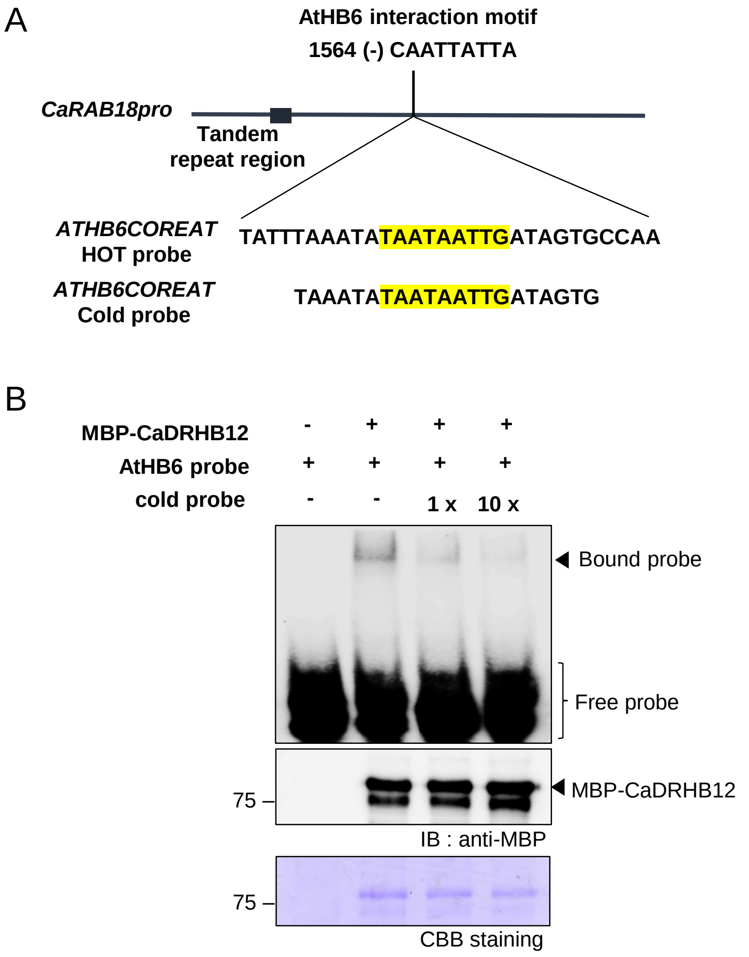

Supplementary Figure S7. EMSA analysis of CaDRHB12 binding to the *CaRAB18* promoter region. (A) Schematic diagram of the *CaRAB18* promoter. The ATHB6COREAT motif is located around the 1555 –1564 bp region. The lower panel shows the use of a biotin-labeled probe and an unlabeled (cold) competitor probe. (B) EMSAs showing biding of CaDRHB12 to AtHB6 interaction motif in the *CaRAB18* promoter in vitro. MBP-CaDRHB12 protein was incubated with the biotin-labeled probe. Binding competition was confirmed using an unlabeled cold probe. Protein input was verified by immunoblotting with an anti-MBP antibody.

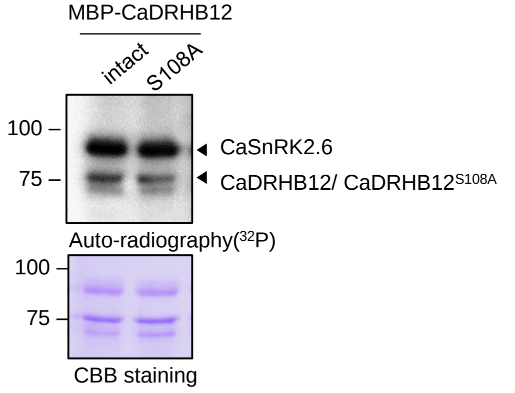

Supplementary Figure S8. *In vitro* phosphorylation of CaDRHB12^{S108A} by CaSnRK2.6. For the *in vitro* kinase assay, CaDRHB12 and CaDRHB12^{S108A} were incubated with CaSnRK2.6. Coomassie brilliant blue (CBB) staining indicated protein input in the kinase assay.

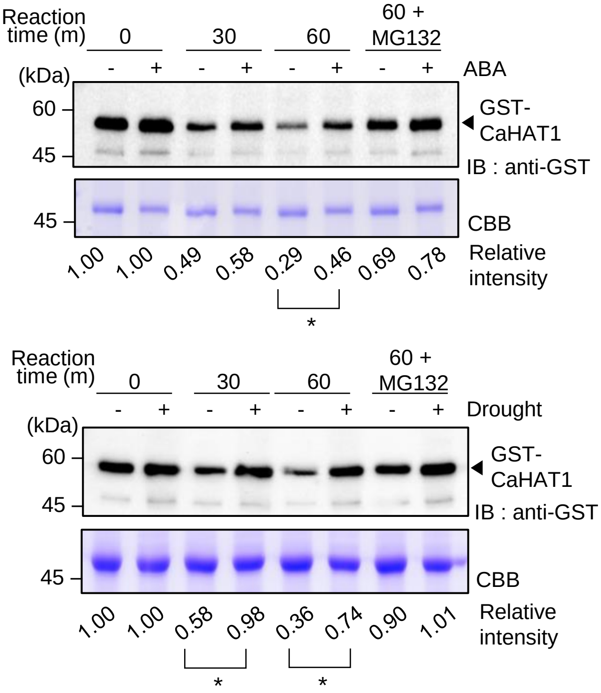

Supplementary Figure S9. Suppression of CaHAT1 protein degradation under ABA exposure and drought stress conditions. For the cell-free degradation assay, crude leaf extracts from pepper leaves treated with 100 µM ABA for 2 h (upper panel) and dried for 6 h (bottom) were mixed with GST-CaHAT1 protein expressed in bacterial cells. The mixtures were incubated at the indicated time points, and MG132 was added as a 26S protease inhibitor. Coomassie brilliant blue (CBB) staining indicated equal loading of the crude extracts. Relative intensities were measured using ImageJ software (NIH), with pre-incubation samples designated as the standard (1.00). All data represent the means ± SD of three independent experiments. Asterisks indicate significant differences between samples (Student's *t*-test; *$P < 0.05$).

Table 1. List of primers used in this study.

Primer name	Primer sequence (5'-3')
**For cloning**	
CaDRHB12-CDSF	Forward: ATGATGATGGAAAAAGAAGAT
CaDRHB12-CDSR	Reverse: TCACTTTTGTCTTGCAAGTGTAG
CaDRHB12 w/o Stop codon	Forward: ACACTTGCAAGACAAAAG AAGGGCGAATTCGACC
	Reverse: GGTCGAATTCGCCCTTCTTTTGTCTTGCAAGTGT
CaDRHB12 a43g_g44c F	Forward: CTCTTTTCTGTTGGAAAGGCTAGGCTGAGGCTCAACCC
CaDRHB12 a43g_g44c R (for S15A)	Reverse: GGGTTGAGCCTCAGCCTAGCCTTTCCAACAGAAAAGAG
CaDRHB12 t202g F	Forward: TCCGAGTTTCTATCTGATGCGGAATCAGGAAATAGAGAC
CaDRHB12 t202g R (for S68A)	Reverse: GTCTCTATTTCCTGATTCCGCATCAGATAGAAACTCGGA
CaDRHB12 a424g_g425c F	Forward: ATCTTCCTCATCGGCAATACCTCGAGAGCTCGCCC
CaDRHB12 a424g_g425c R (for S142A)	Reverse: GGGCGAGCTCTCGAGGTATTGCCGATGAGGAAGAT
CaDRHB12 F1 R	Reverse: TCACTCGCTACGCTTATTTCCACT
CaDRHB12 F2 R	Reverse: TCAACAATTCTCTCCATCTTCCTC
CaDRHB12 F3 R	Reverse: TCATTTTGTCCTAGCTCTCCTATTC
CaDRHB12 F4 R	Reverse: TCACTTTAGTGACCTTAGCTCTTG
CaDRHB12-VIGS-F	Forward: GCCTCAGCCTAAGCTTTCCAAC
CaDRHB12-VIGS-R	Reverse: CATTCGCCGCTGGCATCCT
3FLAGN-CaDRHB12 for overexpression	Forward : ATGGACTACAAAGACCATGACGGTGATTATAAAGATCATGACTACGATTACA AGGATGACGATGACAAGATGATGATGGAAAAAGAAGATCTGG
**For qRT-PCR analysis**	
CaDRHB12-RTF	Forward: GCCTCAGCCTAAGCTTTCCAAC
CaDRHB12-RTR	Reverse: TGGCATCCTGTTCACGTCTATTCC
CaACT1	Forward: GACGTGACCTAACTGATAACCTGAT
	Reverse: CTCTCAGCACCAATGGTAATAACTT
CaOSR1	Forward: ATGGAGGCACAACTGCACCGTC
	Reverse: GGCCCACCATGAACTTCTGCAC
CaRAB18	Forward: ATGTCGCACTACGAGAACCAATATAG
	Reverse: ATCATCCTCAGAGCTGCTGGAGC
CaDRHB1	Forward: GGAATTTCAAAAGAATCATCTAGCA
	Reverse: AGCTAGAAGAGTACTGCATCCAAAA
CaNCED3	Forward: TTAAGGATCTTAAGCGTGGTTATGT
	Reverse: AGATTAGTTCAAGAACGTGAATTGG
AtActin8	Forward: CAACTATGTTCTCAGGTATTGCAGA
	Reverse: GTCATGGAAACGATGTCTCTTTAGT
NCED3	Forward: ACATGGAAATCGGAGTTACAGATAG
	Reverse: AGAAACAACAAACAAGAAACAGAGC
RAB18	Forward: GGAAGAAGGGAATAACACAAAAGAT
	Reverse: GCGTTACAAACCCTCATTATTTTTA
RD29B	Forward: GTTGAAGAGTCTCCACAATCACTTG
	Reverse: ATACAAATCCCCAAACTGAATAACA
DREB2A	Forward: TAGAAAATGCTGGAGGCAAAGTT
	Reverse: TCAGGTTCTGGTCTTGAACTTTCTTT
